# Miniaturization of Aptasensors in Organic Electrochemical Transistors for Sensitivity Enhancement

**DOI:** 10.1021/acs.analchem.6c02236

**Published:** 2026-08-21

**Authors:** Andika Asyuda, Rasheed T. Odunbaku, Luka J. L. Striethorst, Henrique F. P. Barbosa, Andreas Schander, Ramesh Kumar, Dur E. N. Gakhar, Sarah Bornemann, Rachel E. Armstrong, Björn Lüssem

**Affiliations:** † Institut für Mikrosensoren, Aktoren, und -Systeme (IMSAS), 9168 Universität Bremen, Bremen 28359, Germany; ‡ Light Technology Institute, Karlsruhe Institute of Technology, Karlsruhe 76131, Germany; § 441161 InnovationLab GmbH, Heidelberg 69115, Germany; ∥ OnePlanet Research Center, Wageningen 6708 WH, The Netherlands; ⊥ imec, Wageningen 6708 WH, The Netherlands; # MAPEX Center for Materials and Processes, Universität Bremen, Bremen 28334, Germany

## Abstract

Aptasensors have received intense interest because of their selectivity and synthetic flexibility. Aptamer bioreceptors were recently combined with organic electrochemical transistors (OECTs) to increase the signal strength. Although promising, a lack of fundamental understanding of the scaling of these OECT-based aptasensors makes targeted optimization of these devices challenging. Here, we study the influence of the gate electrode area on sensitivity, detection range, and selectivity systematically. To increase the ratio between gate and channel area further, we, for the first time, combine the aptasensor with a vertical OECT (VOECT), yielding a sensitivity as high as 0.3 mA/dec. Furthermore, the response of the OECT aptasensor is already significant at gate voltages close to 0 V, thus avoiding undesired electrochemical processes such as gold oxidation or radical oxygen formation. Overall, the combination of OECT and aptasensor serves as an important step toward a miniaturized biosensor with amplified sensitivity and continuous sensing capability. The present study not only extends our understanding of gate scaling on the sensor response but also provides design rules to reach very high sensitivity at low operational voltages.

## Graphical abstract

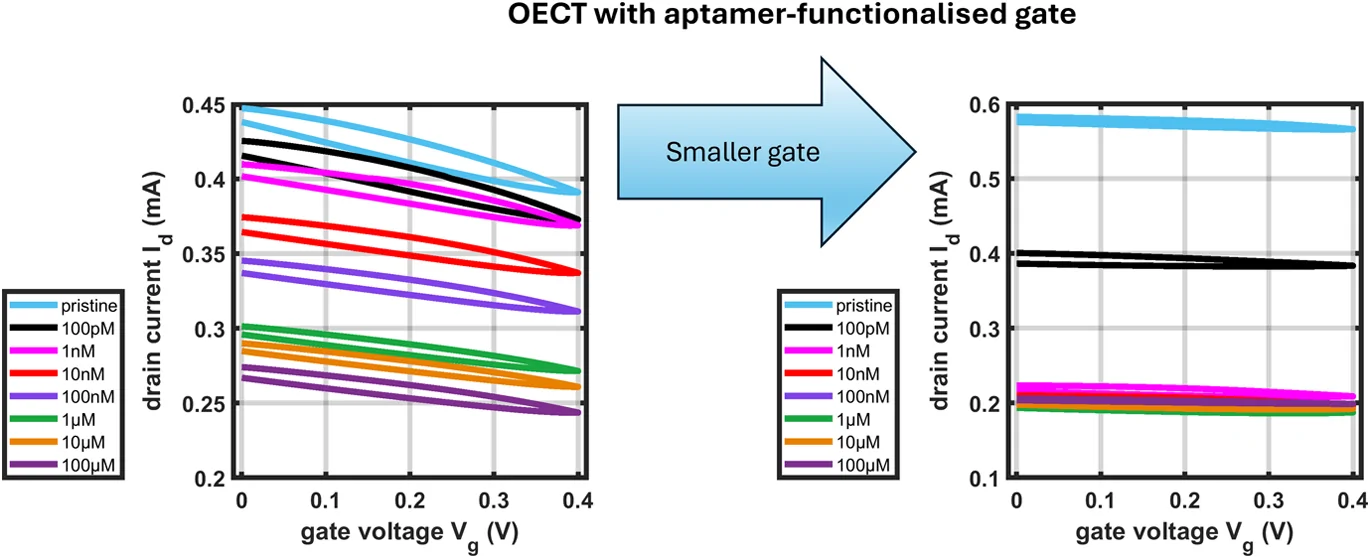

## Introduction

Point-of-care (POC) diagnostics is gaining importance, mostly driven by the necessity for early and easy detection of various diseases, which enables a higher treatment success rate.^1–3^ However, POC diagnostics heavily rely on portable and highly sensitive biosensors. Aptamers are one of the most promising candidates for biosensor receptors due to their high affinity and specificity to the targeted bioanalyte, which equals or even exceeds the affinity of immunosensors, the current gold bioreceptor standard.^4,5^ Additionally, aptamers can be produced at low cost with high reproducibility and can be modified with anchor groups (e.g., thiol groups) and signal tags, facilitating the coupling of various detection techniques with aptamers.^6,7^

A change in aptamer conformation due to an interaction with the analyte is mostly detected optically or electrochemically.^8–12^ A redox reporter is typically included in a system of electrochemical detection, either in the electrolyte or attached to the aptamers. Electrochemical interrogation of the aptamer sensor, or aptasensor, is highly preferred since it is compatible with portable devices, microelectronics, and miniaturization.^2,13^ Usually, a classical setup of three electrodes is used for electrochemical sensing, consisting of working, counter, and reference electrodes.^14,15^

Unfortunately, it is challenging to miniaturize an electrochemical aptasensor in the classical three-electrode setup, since as the area of the working electrode decreases, the measured current diminishes as well.^16^ Interestingly, Dastidar et al. have shown that as the area of the working electrode decreases, thus fewer adsorbed aptamers on the electrode, the relative change in current, i.e., normalized sensitivity, increases.^17^ This suggests that the measured current and the sensitivity have an opposite dependence with respect to the area of the working electrode and cannot be simultaneously increased in a classical setup of three electrodes by varying the area of the working electrode only.

Electrochemical aptasensors, especially the ones using thiol chemistry to immobilize molecules on gold substrates,^18,19^ are also restricted in terms of the voltage that can be applied without degrading the sensor. Usually, the voltage window is limited to −0.3 and +0.1 V (±0.1 V) vs Ag/AgCl.^20–22^ A higher absolute voltage will trigger gold oxidation or radical oxygen formation, leading to the loss of aptamer molecules. Several approaches have been explored to increase the stability of aptasensors, for example, by protecting the gold surface with a dense monolayer,^20–24^ using alternative binding chemistry for immobilization other than thiol,^25,26^ or alternative substrates other than gold.^25,27,28^

As an alternative to classical three-electrode setup, aptasensors have been used as the gate electrode of an organic electrochemical transistor (OECT).^16,29–34^ OECTs are known for their inherent signal amplification. This amplification is caused by the working mechanism of OECTs, where a small change in the injection of ions into a so-called organic mixed ionic-electronic conductor (OMIEC) leads to a large variation in the conductivity of OMIECs. When a voltage is applied to the OMIEC via the source and drain electrodes, the variation in conductance results in a large variation in the drain current.^6,13,35^

Although OECT-based aptasensors were presented previously, there is still a lack of fundamental understanding of the scaling of these devices, making a targeted optimization challenging. For example, for devices without aptamer functionalization, a systematic optimization of OECTs toward higher sensitivity was reported by Cicoira et al. and Bernards et al.^36,37^ It was shown that OECTs with smaller gate electrode area are more sensitive, as more voltage drops at the gate-electrolyte interface compared to the voltage drop at the electrolyte-channel interface.^37^

Here, we study the influence of the gate electrode area on sensitivity, detection range, and selectivity of OECT-based aptasensors systematically. To increase the ratio between gate and channel area further, we, for the first time, combine the aptasensor with a vertical OECT (VOECT), yielding a sensitivity as high as 0.3 mA/dec. Using this strategy, the aptasensor response is not only amplified but can also be tuned in terms of the detection range and sensitivity, according to the requirements of medical diagnostics.

Thrombin and thrombin-sensitive aptamers with a specific sequence of 29 bases, or TBA-29 for short, are chosen in the current study. Thrombin is a vital enzyme in blood coagulation, holding a key role in the conversion of fibrinogen to fibrin and regulating several other factors to prevent excessive blood loss in the event of blood vessel damage.^38,39^ Thrombin is usually absent from the human body and is only activated at the site of injury in trace quantities (pM to nM range) and up to μM concentrations during the coagulation process.^40,41^ Thrombin imbalances can lead to various medical conditions, such as heart attack, stroke, liver disease, deep vein thrombosis, leukemia, pulmonary embolism, and central nervous system disease.^42,43^ Monitoring of thrombin levels is particularly important in several specific cases, such as during pregnancy and surgery, to prevent deep vein thrombosis.^42^

Overall, the integration between OECT and aptasensor serves as an important step toward a miniaturized biosensor with amplified sensitivity and continuous sensing capability. The present study not only extends our understanding of gate scaling on the sensor response, but also provides design rules to reach very high sensitivity at operational voltages (|*V*_*g*_ |+|*V*_*d*_ |) as low as +0.2 V. This low operational voltage is highly important for a continuous monitoring of bioanalyte and to avoid degradation of an aptasensor due to electrochemical investigation.^20–22,44^

## Experimental Section

### Materials and Chemicals

Phosphate-buffered saline (PBS) tablets, thrombin from bovine plasma (50 NIH units/mg), magnesium chloride MgCl_2_ powder, tris­(hydroxymethyl)-aminomethanhydrochlorid Tris HCl (purity ≥ 99%), ethylenediaminetetraacetic acid EDTA disodium salt solution (0.5 M in H_2_O), potassium hexacyanoferrate­(II) trihydrate K_4_Fe­(CN)_6_·3H_2_O (purity ≥ 99.95%), 6-mercapto-1-hexanol MCH (97% assay), dodecylbenzenesulfonic acid DBSA solution (weight percentage 70% in isopropanol), (3-glycidyloxypropyl)­trimethoxysilane GOPS (≥98% assay), and tris­(2-carboxyethyl) phosphine hydrochloride TCEP powder were purchased from Merck. Potassium hexacyanoferrate­(III) K_3_Fe­(CN)_6_ (purity ≥ 96%) was purchased from VWR Chemicals. Clevios PH 1000 was purchased from Heraeus Epurio. Both thrombin-sensitive and random 29-mer oligo were purchased from Integrated DNA Technologies (IDT) with thiol modification at the 5′ end and HPLC purification. The sequence of thrombin-sensitive oligo TBA-29 is/5ThioMC6-D/AG TCC GTG GTA GGG CAG GTT GGG GTG ACT, while the random 29-mer oligo is/5ThioMC6-D/AA CGA TCT CTT GCG AGC GCT TGA CCA CCT. Note that the implementation of TBA-29 as a thrombin aptasensor, using a classical three-electrode setup, has been shown in the literature, but there are no reports before our work using TBA-29 to fabricate an OECT-based aptasensor for thrombin.^15,45,46^

### Oligo Sample Preparation

1.0× PBS solution was made by dissolving 1 PBS tablet in 200 mL of DI water. 500 μM oligo solution was prepared in suspension buffer from thiolated and lyophilized oligo and 10 mM Tris HCl 1 mM EDTA solution. If not used immediately, the solution was stored at −20 °C. 100 μM oligo solution was prepared by diluting the 500 μM oligo solution with 0.5 mM MgCl_2_ in 1.0× PBS and then stirred at 95 °C for 5 min. Afterward, the solution was cooled at room temperature for 15 min. The oligo solution was then mixed with the 20 mM TCEP solution in PBS and left at RT for 60 min. Thereafter, the solution was used for an overnight incubation of either a gold stripe or gold electrode of an OECT.

After incubation in an oligo solution, the samples were washed with 1.0× PBS, dried with compressed air, and then incubated in 10 mM MCH in 10 mM PBS for 1 day for the gold stripe samples or 15 min for the OECT samples. After incubation in MCH solution, the samples were washed with 10.0× PBS, dried with compressed air, and stored before measurement.

### Lateral OECT (LOECT) Fabrication

Silicon wafers were used as substrates for LOECT fabrication. A 0.1 vol % aqueous solution of 3-aminopropyltriethoxysilane (APTES, purity 99%) was applied to these wafers to improve the adhesion of the subsequent layer. Polyimide (U-Varnish S, UBE Corporation) was then deposited by spin coating and baked in vacuum, creating a 5 μm thick insulation layer and an adhesion layer for the gold layer. Gold was then applied by magnetron DC sputtering (Pro Line PVD 75, Kurt J. Lesker Company Ltd.), and later patterned by photolithography (AZ 1518, MicroChemicals GmbH) and wet etching (0.1 M iodine solution). Next, the wafer was subjected to a short plasma treatment with oxygen reactive ion etching (RIE) to increase the roughness of the surface of the polyimide layer, thus improving the adhesion between this layer and the next layer. The next layer was a diluted polyimide in *N*-methyl-2-pyrrolidone in a 1:1 mass ratio to form a 300 nm thick polyimide film to passivate the contact paths. Afterward, a third polyimide coating was applied to form another 5 μm thick film that serves as a sacrificial layer to later pattern the active channel. The second and third polyimide layers were then subjected to a photolithography step (AZ 10XT, MicroChemicals GmbH) and RIE oxygen plasma treatment (STS ICP) to open the active areas of the transistor so that the active material could be deposited by subsequent deposition.

Before PEDOT:PSS deposition on the active channel region, the gate electrode was protected by an aluminum foil to keep it clean. For PEDOT:PSS deposition, a mixture of 2 mL of poly­(3,4-ethylenedioxythiophene)-polystyrenesulfonate (PEDOT:PSS) with 500 μL of ethylene glycol (EG), 5 μL dodecylbenzenesulfonic acid (DBSA), and 24.2 μL (3-glycidyloxypropyl) trimethoxysilane (GOPTS) was prepared and deposited by spin coating. Before deposition, the solution was filtered through a PTFE filter with a pore size of 0.45 μm. The substrate was then spun at 1000 rpm for 60 s. Later, the samples were dried on a hot plate at 120 °C for 15 min. In the last step, the sacrificial layer was removed, resulting in films with a thickness of 250 nm between source and drain contacts only.

### VOECT Fabrication

Fabrication of VOECT follows a previously published procedure.^47–49^ VOECTs were fabricated on silicon wafers coated with a 500 nm thick silicon oxide layer. A 5 μm thick polyimide layer (U-Varnish S, UBE Corporation) was spin-coated and cured using a vacuum hot plate for an insulation layer. A 250 nm thick gold layer was deposited using magnetron DC sputtering (Pro Line PVD 75, Kurt J. Lesker Company Ltd.), and patterned using photolithography (AZ 1518, MicroChemicals GmbH) and wet chemical etching (Au etch 200, NB Technologies GmbH) to create the source electrodes. Then, a 300 nm thin layer of polyimide was spin-coated and cured to insulate the source electrodes and to define the channel length of the VOECT. Afterward, the second gold layer was deposited and patterned using the same processes as described above to fabricate the ring-shaped drain electrodes. To insulate the conducting paths and define the area for PEDOT:PSS electropolymerization, another 5 μm thick polyimide layer was spin-coated and cured. To improve the adhesion between the polyimide layers significantly, a short (30 s) oxygen RIE plasma treatment (STS ICP) was performed directly before the coating of polyimide. The top insulation layer was then structured together with the thin polyimide layer using photolithography (AZ 10XT, MicroChemicals GmbH) and oxygen RIE plasma (STS ICP). After wafer dicing, the electropolymerization process was performed. For the electrodeposition of PEDOT:PSS, a monomer solution of 10 mM EDOT (3,4-ethylenedioxythiophene, Sigma-Aldrich) and 2 wt % of NaPSS (poly­(sodium 4-styrenesulfonate), MW 70000, Sigma-Aldrich) dissolved in DI water was used. A constant current density of 5 μA/mm^2^ was applied between the samples and a platinum counter electrode. For better homogeneity of the coating, the monomer solution was stirred slowly. The source and drain electrodes were coated simultaneously with PEDOT:PSS for 60 s. After coating, the samples were cleaned in DI water and dried with nitrogen.

### OECT and OECT Aptasensor Characterization

The LOECTs were characterized using a Keithley 4200A-SCS parameter analyzer, a custom-made MBox switch matrix, and PDMS well to hold the electrolyte and thrombin solution. The VOECTs were characterized using a Keithley 2612B system source meter. For aptasensor characterization, *V*_d_ is held at −0.2 V and *V*_g_ is scanned between 0 and 0.4 V, unless otherwise stated. To measure the response of OECT to the analyte, the analyte is incubated with OECT for 30 min prior to measurement.

We tested both lateral and vertical OECT-based aptasensors at least twice for each gate-to-channel area ratio across different chips. We noted that both normalized and absolute sensitivities of the sensor are reproducible, while the linear range of detection can vary by 1 order of magnitude.

### Electrochemical Characterization of Aptamer

Cyclic voltammetry (CV), differential pulse voltammetry (DPV), and electrochemical impedance spectroscopy (EIS) were conducted with the Ivium-n-Stat electrochemical station. For DPV and CV, each measurement was repeated 3 times. For DPV, pulse time, pulse amplitude, E-step, scan rate, and voltage range were 200 ms, 50 mV, 10 mV, 20 mV/s, and −0.2 to 0.6 V, respectively. For CV, the scan rate and voltage range are 50 mV/s and between −0.2 and 0.6 V, respectively. EIS measurements were performed at open-circuit voltage (OCP), signal amplitude 5 mV, and frequency range 60 kHz–0.1 Hz. All the electrochemical measurements were performed at room temperature using a 1:1 mixture of 10 mM K_3_[Fe­(CN)_6_]/K_4_[Fe­(CN)_6_] as electrolyte, and PBS 1.0× as supporting electrolyte. A platinum film on glass with an area 1 cm × 5 mm is used as the counter electrode. A gold stripe on glass with a titanium adhesion layer with an area 5 mm × 5 mm is used as a working electrode and substrate for the aptamer self-assembled monolayer sample. As a reference, an electrode model SE11 from Sensortechnik Meinsberg is used. To characterize the sensor performance, thrombin was introduced into the electrolyte and measured after 30 min of incubation. The limit of detection was calculated on the basis of the average and standard deviation of the random oligo according to the formula average + 3 × standard deviation­(σ).

### Electrochemical Characterization of the PEDOT:PSS Film

EIS was conducted with an Ivium-n-Stat electrochemical station at room temperature and open-circuit voltage (OCP), using a signal amplitude of 50 mV, a frequency range of 100 kHz–0.1 Hz, and an aqueous NaCl solution 0.1 M as an electrolyte. A platinum film on glass with an area of 1 cm × 5 mm is used as the counter electrode. A gold stripe on glass with a titanium adhesion layer and an area of 2.5 mm × 2.5 mm is used as a working electrode and substrate for PEDOT:PSS deposition, either by spin coating or electropolymerization. As a reference, an electrode model SE11 from Sensortechnik Meinsberg is used.

### Transient Measurement of LOECT-Based Aptasensor

Transient measurements were performed with the Tektronix mixed-signal oscilloscope MSO46 with a home-built amplifier to convert and amplify measured currents to voltage signals. For measurement, *V*_d_ is held at −0.2 V, while *V*_g_ is pulsed between 0 and 0.4 V, each for 5 s, for a total duration of 30 s. The collected *I*_d_ and *I*_g_ are smoothed using a moving average filter. For statistical analysis, the data around the voltage change (±0.1–2 s) is omitted to ensure that a steady-state response of both *I*_d_ and *I*_g_ is considered. The limit of detection was calculated on the basis of the median absolute deviation (σ) of the drain current (on or off state) of the unexposed sample according to the formula 3 × σ.

## Results and Discussion

### Performance of Thrombin Aptasensor in Standard Electrochemical Measurements

To show the proper function of the aptasensor, the response of self-assembled films of TBA-29 is characterized using standard voltammetry, i.e., in a classical three-electrode setup, before combining them with OECTs later on. The working electrode is fabricated by immersing a thin gold film, processed as described in the Experimental Section, in a TBA-29-thiol solution overnight, followed by immersion in a 6-mercapto-1-hexanol (MCH) solution. MCH is commonly used to passivate parts of the gold surface that were not covered by aptamer molecules during the first assembly step and to prevent biofouling of the aptasensor by the analyte.^18^ Solvated ferro-/ferricyanide in PBS 1.0× buffer solution is used as the electrolyte.

To monitor the interaction of aptamers with an analyte, differential pulse voltammetry (DPV) is commonly used.^10,14^ Any conformational change in the aptamer due to its interaction with the analyte changes the electron transfer rate from the redox reporter to the gold substrate,^50^ which is then seen as a change in the current amplitude of the pulsed voltammogram. In comparison to cyclic voltammetry (CV), a higher sensor sensitivity and lower limit of detection (LOD) are usually found. This higher sensitivity is often explained by a smaller contribution of capacitive charging currents in pulsed measurements, which allows us to distinguish more clearly any change in Faradaic currents.^51,52^

The result of the DPV measurement of a pristine aptasensor is shown in Figure 1A. An oxidation peak is seen at ≈0.23 V, indicating unimpeded mass transport of ferro-/ferricyanide to the surface of the gold electrode, despite the chemisorbed TBA-29 and MCH on the gold. It can be observed that by increasing the concentration of thrombin in the electrolyte, and therefore increasing the interaction between thrombin and TBA-29, the amplitude of the oxidation current peak systematically decreases.

**Figure 1:**
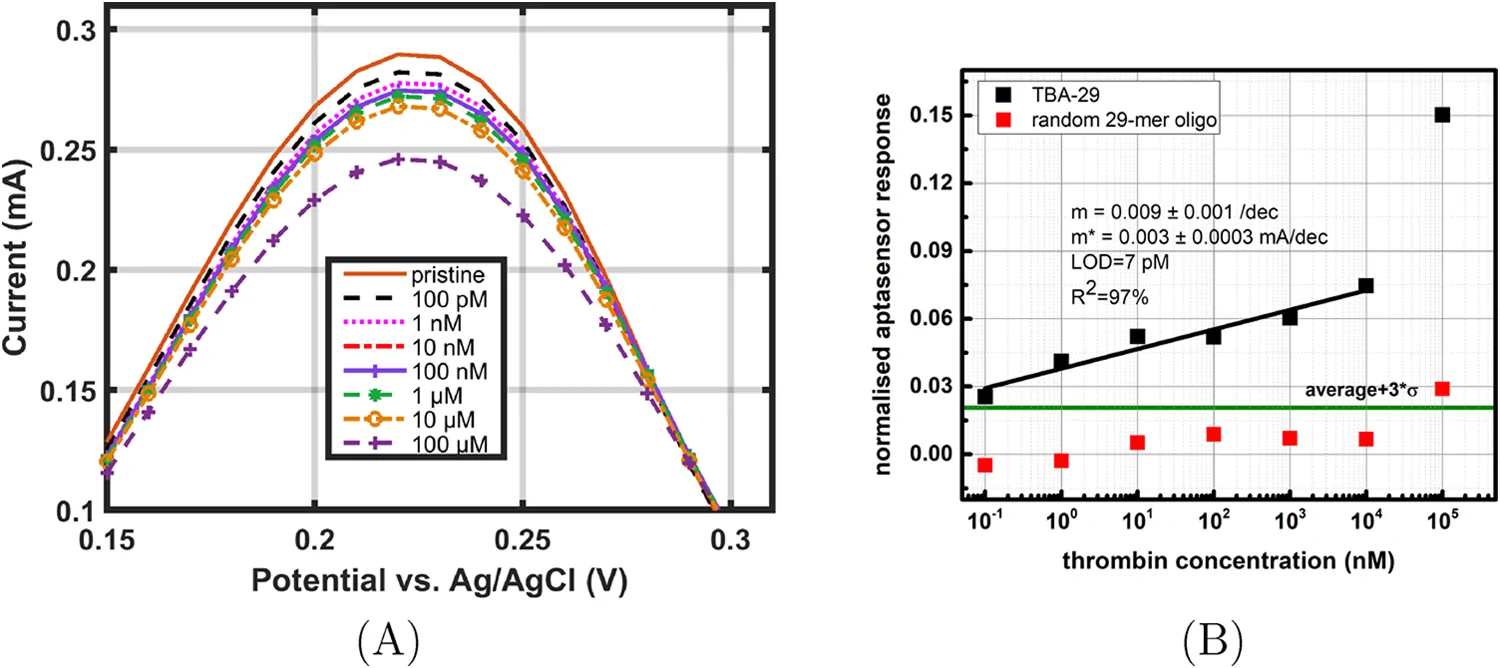
Standard aptasensor response using differential pulse voltammetry (DPV). (A) DPV of TBA-29 aptasensor on a gold measured in a standard electrochemical cell consisting of three electrodes. The sample is exposed to increasing concentrations of thrombin, varying between 100 pM and 100 μM. (B) Normalized response of the aptasensor extracted from the DPV measurements shown in (A) and Figure S1, for the TBA-29 aptasensor and the control experiment using a random 29-mer oligo, respectively. The normalized response is calculated using eq 1 and the current of the DPV oxidation peak.

As a control experiment, an oligomer of 29 bases with a random sequence (random 29-mer oligo), which should not interact with thrombin, is used instead of TBA-29. As shown in Figure S1, this random oligomer sample exhibits an almost unchanged oxidation current in the DPV measurement, regardless of thrombin exposure.

To determine the sensitivity of the aptasensor and its linear detection range, the normalized response of both TBA-29 and random 29-mer oligo is calculated by

1 $$\frac{\Delta I}{I_{0}}=\frac{I_{\mathrm{pris}}-I_{\mathrm{thr}}}{I_{\mathrm{pris}}}$$

where *I*_thr_ is the oxidation peak current of the sample after exposure to thrombin and *I*_pris_ is the oxidation peak current of the pristine, i.e., unexposed sample.

The comparison of the normalized response between TBA-29 and the random oligo is shown in Figure 1B. Although the sensor response for TBA-29 changes linearly with the concentration of thrombin, the response of the random oligo is almost independent of the thrombin concentration. The normalized (*m*) and absolute sensitivity (m*) of the TBA-29 aptasensor can be obtained from the slope of the linear fit shown in Figure 1B and amount to 9 × 10^–3^/dec and 3 × 10^–3^ mA/dec, respectively.

The limit of detection (LOD) of the aptasensor is calculated from the normalized response of random 29-mer oligo, according to

$$\mathrm{LOD}=10^{\frac{({\left(\frac{\Delta I}{I_{0}}\right)}_{\mathrm{random}}+3\sigma )-B}{A}}$$ 2

where ${\left(\frac{\Delta I}{I_{0}}\right)}_{\mathrm{random}}$ and σ are the average and standard deviation of the normalized response of the random 29-mer oligo, respectively, while *A* and *B* are the y-intercept and gradient of the linear fit of the aptamer response, respectively. The LOD is shown in Figure 1B as the intersection between the green line and the calibration curve, viz. 7 pM.

The results obtained by DPV are confirmed using electrochemical impedance spectroscopy (EIS) shown in the Supporting Information (Figures S2A–D and S3A,B) and CV (Figures S4 and S5).^14,53^ The EIS spectra are fitted with an equivalent circuit shown in Figure S3A, and the results are presented in Table S1. Note that the geometrical capacitance of adsorbed TBA-29 and MCH on gold is 1 μF for the sample area of 25 mm^2^.

Our results are in agreement with electrochemical thrombin aptasensors reported in the literature (cf. Table S2). Note that the LOD and sensitivity of an aptasensor vary with the electrode area, which can explain the wide range of reported LODs in the literature.^17^ The area of our working electrode (25 mm^2^) is comparatively large, allowing more aptamers to be adsorbed on the electrode, which consequently leads to a lower relative contribution of each aptamer to the total ionic current, lower normalized sensitivity, and higher LOD. However, at the same time, the linear detection range of our aptasensor is wider, most likely caused by the large working electrode as well.^17^

### Functionalizing the Gate Electrode of Lateral OECTs with Aptamers

Although electrochemical interrogation of a thrombin aptasensor using a classical three-electrode setup, for example, DPV, CV, and EIS, yields a consistent signal that is proportional to thrombin concentration, the signal decreases for devices with smaller dimensions. To increase the response of the aptasensor, both TBA-29 or random oligo and MCH are chemisorbed onto the Au gate electrode of a lateral OECT (LOECT) by thiol bonding, as illustrated in Figure 2A. The OECT is processed according to the recipe provided in the experimental details. Each chip consists of 3 LOECTs with identical dimensions of the PEDOT:PSS channel (500 μm × 500 μm). There is a 10 μm overlap between PEDOT:PSS and source/drain contacts, which is not counted toward the channel length of 500 μm. The channel is structured by peeling the lift-off layer after PEDOT:PSS is spin-coated on the device. The area of the gold gate is varied with respect to the channel area, resulting in ratios of 10:1, 1:1, and 1:10, as shown in Figure 2B. PBS 1.0× is used as the electrolyte.

**Figure 2:**
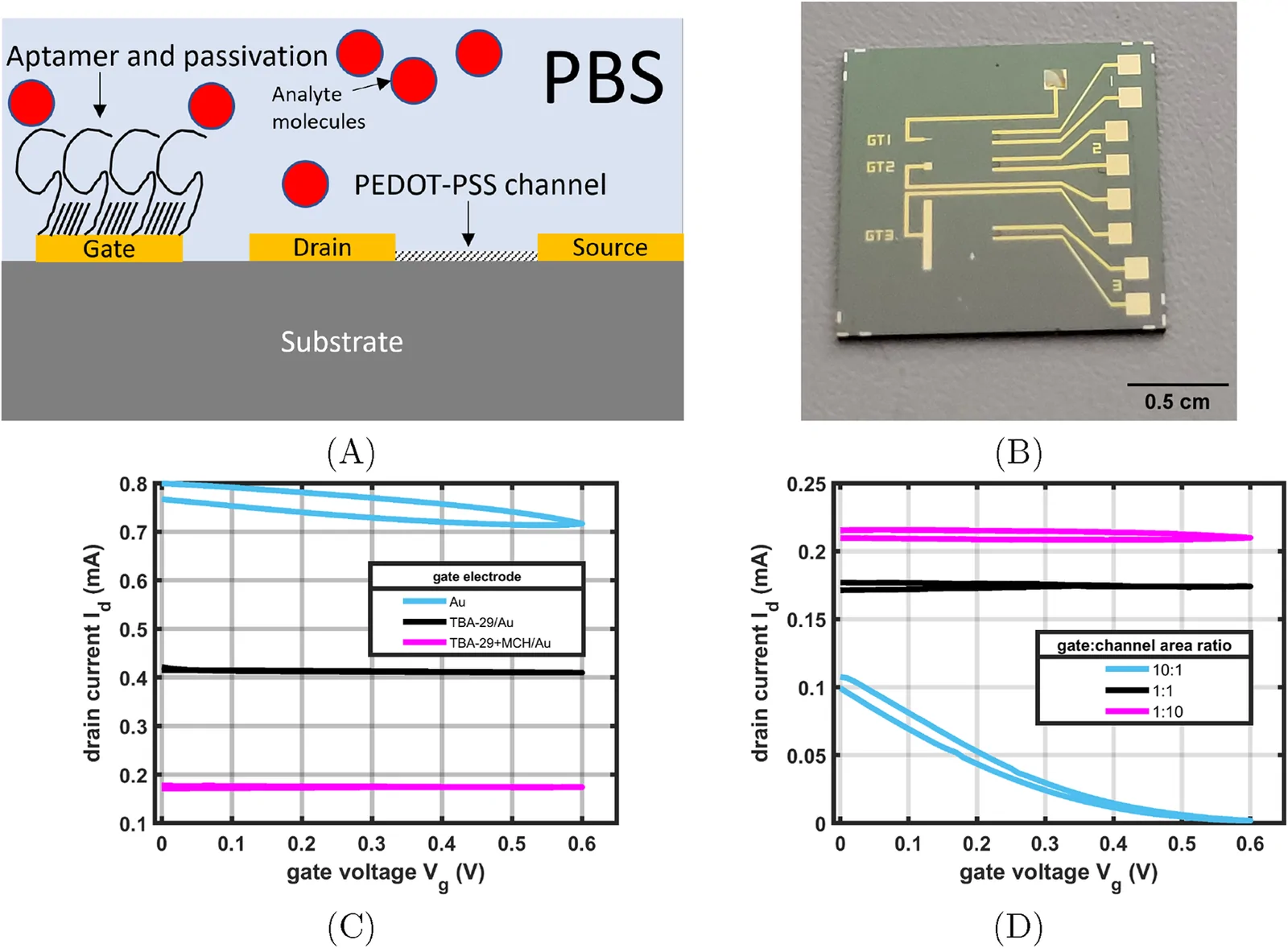
Functionalization of the gate of a lateral OECT with TBA-29 and MCH SAMs. (A) Illustration of a lateral OECT (LOECT), in which source, drain, and gate electrodes are prepared on the same plane. A channel of PEDOT:PSS is deposited between a source and drain electrodes. TBA-29/random oligo and MCH molecules are adsorbed on the gate electrode. PEDOT:PSS channel and gate electrode are covered with phosphate-buffered saline (PBS) 1.0× solution as both electrolyte and buffer solution for thrombin. (B) Photo of a chip, consisting of 3 LOECTs, consisting of identical channel area (500 μm × 500 μm) and gate-to-channel area ratio of 10:1, 1:1, and 1:10. (C) Transfer characteristics of LOECTs with equal area of gate and channel (500 μm × 500 μm), before assembly of the aptamer, i.e., measured with clean gold gate electrodes, after adsorption of TBA-29, and following the adsorption of MCH on gate electrode. (D) Transfer characteristics of LOECTs with constant channel dimensions and increasing area of the gate electrode with adsorbed TBA-29 and MCH, effectively varying the gate-to-channel area ratio from 10:1 to 1:1 and 1:10. For all measurements, the drain voltage *V*_d_ is kept at −0.2 V.

The transfer characteristics of the LOECT with equal gate and channel area, before and after assembly of the SAM consisting of TBA-29 and MCH on the gate, are shown in Figure 2C. The absolute drain current *I*_d_ decreases significantly after molecular adsorption on the Au gate electrode. *I*_d_ at *V*_g_ = 0 V, referred to in the following as *I*_d‑on_, drops from 0.8 mA to 0.4 mA and 0.2 mA for the clean Au gate, TBA-29 on the Au gate, and TBA-29/MCH on the gate electrode, respectively. This behavior is also seen in the OECT output characteristic shown in Figure S6A.

In all devices, in particular, in devices with adsorbed TBA-29 or MCH on the gate, the ON/OFF ratio is small. This effect can be explained by the limited voltage range and low capacitance of the gate in comparison to the channel. According to the model of Bernards et al.,^37,54^ the gate electrode/electrolyte/channel junction can be modeled as a series connection of two capacitors—the capacitance between the gate electrode and the electrolyte *C*_*g*_ and the channel capacitance *C*_ch_. Following this approximation, only the fraction $V_{\mathrm{ch}}=V_{g}\frac{C_{g}}{C_{\mathrm{ch}}}$ drops across the channel.

From the EIS measurements of the TBA-29-/MCH-covered gold substrate and the spin-coated PEDOT:PSS channel (cf. Figures S2A and S6B) and the modeling of the EIS spectrum by the equivalent circuit shown in Figures S3A and S7A, one can extract the gate and channel capacitances, *C*_g_ and *C*_ch_, respectively. From Table S1, it can be inferred that the adsorption of TBA-29 and MCH on the 500 μm × 500 μm gold electrode reduces its capacitance to *C*_g_ = 1 × 10^–2^ μF, due to the length of the aptamer. Considering the volumetric capacitance of PEDOT:PSS (50 F/cm^3^) and the thickness of the spin-coated PEDOT:PSS channel (200 nm), the capacitance of the gate electrode is smaller than the capacitance of PEDOT:PSS channel (*C*_ch_ = 2.5 μF). Therefore, the capacitance ratio *C*_g_/*C*_ch_ drops to 0.004, i.e., almost all applied gate potential drops at the gate electrode/electrolyte interface, inducing a very low *I*_d_ modulation, as seen in Figure 2C.

To increase modulation in the OECT, the capacitance ratio *C*_g_/*C*_ch_ can be enlarged by increasing the area of the gate electrode, as shown in Figure 2D. Here, TBA-29 and MCH are adsorbed on the gate electrodes with varying areas, while the dimensions of the PEDOT:PSS channel are kept constant (500 μm × 500 μm), resulting in gate-to-channel area ratios of 10:1, 1:1, and 1:10. These ratios result in capacitance ratios between the gate electrode and the channel of 0.04, 0.004, and 0.0004. As shown in Figure 2D, the modulation of the drain current increases for the OECT with a gate electrode area larger than the channel area. The OECT with a gate electrode area 10 times smaller than the channel area does not exhibit any drain current modulation. The corresponding gate current *I*_g_ is shown in Figure S7B.

### Detection of Thrombin with Lateral OECT Aptasensors

In the previous section, it was shown that TBA-29 responds to a change in exposure to thrombin, depending on the analyte concentration, using ferro-/ferricyanide electrolyte with PBS as supporting electrolyte. A similar effect is seen in LOECTs using only PBS as electrolyte, as shown in Figure 3A for a device with a gate-to-channel area ratio of 1:1. The associated *I*_g_ vs *V*_g_ is shown in Figure S8. Note that the range of *V*_g_ is limited to 0.4 V to ensure the stability of the aptamer/MCH SAM over an extended period to be able to study the response of TBA-29 to multiple concentrations of thrombin.^55^

**Figure 3:**
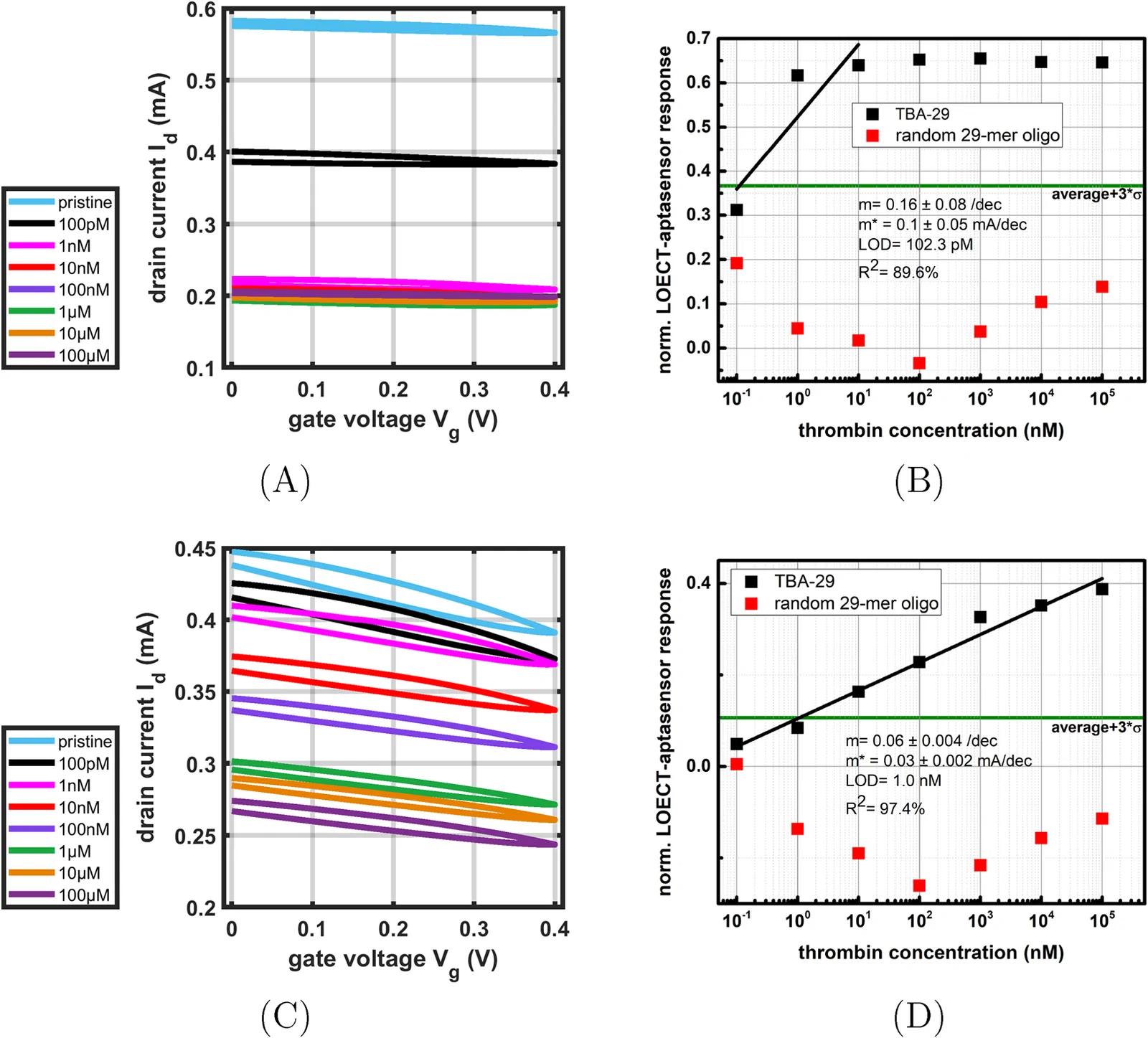
Response of LOECT-based aptasensors to varying thrombin concentrations. (A, C) Transfer characteristics and (B, D) calibration curves of LOECTs with adsorbed TBA-29 and MCH on gold gate electrodes with gate-to-channel area ratio of (A, B) 1:1 and (C, D) 10:1. The drain voltage *V*_d_ is kept constant at −0.2 V. The normalized response of LOECT with adsorbed TBA-29 (from panels (A) and (C)) and random 29-mer oligo (from Figures S9A and S11A) on the gate are calculated using eq 3.

As depicted in Figure 3A, the drain current of the LOECT drops consistently with increasing thrombin exposure. Most importantly, the response is already significant at a gate voltage *V*_g_ = 0 V. The normalized response of the device to thrombin is calculated by

3 $$\frac{\Delta I}{I_{0}}=\frac{I_{d‐\mathrm{on}}\left(\mathrm{pris}\right)-I_{d‐\mathrm{on}}\left(\mathrm{thr}\right)}{I_{d‐\mathrm{on}}\left(\mathrm{pris}\right)}$$

where *I*_d‑on_(pris) and *I*_d‑on_(thr) are the on-drain current (*I*_d_ at *V*_g_ = 0 V) of the OECT before and after exposure to thrombin, respectively. The normalized response of the TBA-29-functionalized LOECT is compared with the random-oligo-functionalized LOECT with the same dimensions (Figure S9A for *I*_d_ and Figure S9B for *I*_g_) in Figure 3B. While a consistent response of the LOECT functionalized with TBA-29 is seen between 100 pM and 10 nM thrombin exposure, the LOECT functionalized with random oligo exhibits a much smaller response without a consistent trend. The LOD, normalized, and absolute sensitivity of the LOECT-based aptasensor for thrombin are 102.3 pM, 0.16/dec, and 0.1 mA/dec, respectively.

The corresponding results for an OECT with an increased gate-to-channel area ratio of 10:1 are shown in Figure 3C, with *I*_g_ vs *V*_g_ shown in Figure S10. As a control experiment, the transfer characteristic of LOECTs with adsorbed random 29-mer oligo and MCH on the gate electrode is shown in Figure S11A for *I*_d_ vs *V*_g_ and Figure S11B for *I*_g_ vs *V*_g_. The calibration curve of the aptasensor is shown in Figure 3d. Similar to Figure 3B, the response of the LOECTs with a gate-to-channel area of 10:1 is already strong at *V*_g_ = 0 V. The response increases linearly with the concentration of thrombin, with normalized and absolute sensitivities *m* and m* of 0.06/dec and 0.03 mA/dec, respectively. Note that, despite the LOECT-based aptasensor with a larger gate area exhibiting a larger ON/OFF ratio than the device with a smaller gate, its normalized sensitivity is lower, which agrees with the observation of Dastidar et al. in a classical setup of three electrodes.^17^

Additionally, TBA-29-functionalized LOECTs with a gate-to-channel area ratio of 1:10 are tested and discussed in the Supporting Information (cf. Figures S12 and S13A–D).

Note that, regardless of the ratio between the gate electrode and the channel area, the sensitivity of our OECT-based aptasensor is higher than that of most of the reported OECT-based aptasensors without a redox reporter tag and a larger gate electrode.^56,57^ Recently, Burtscher et al. demonstrated a detection of interleukin-6 using an OECT-based aptasensor, with an equal area between the PEDOT:PSS channel and the PEDOT:PSS gate electrode and frequency-dependent measurements, which exhibits comparable sensitivity to our LOECT with a gate-to-channel area ratio of 1:10.^29^ The small difference in response can come from the reactivity of the aptamer itself with the respective analyte. The detection of thrombin using an OECT-based aptasensor, but with a different aptamer than the one used in our work, was shown by Peruzzi et al. In this work, a thrombin sensor with a higher sensitivity than our LOECTs is shown by interfacing aptamers, nanoparticles, graphene, and redox reporters in an OECT.^33^ Our experiments show that not only an OECT-based aptasensor with a capacitance ratio *C*_g_/*C*_ch_ below 4 can be used for aptasensing but also that the smaller gate can lead to a biosensor with high sensitivity.^32,58,59^

### Detection of Thrombin with Voltage Pulses

As an alternative to the full transfer characteristic sweeps discussed above, LOECTs can also be operated by short gate voltage pulses. In Figure 4A, the response of an LOECT-based aptasensor with a gate and channel ratio of 1:1 is shown. The sample is exposed to alternating gate voltage pulses *V*_g_, switched between 0 and 0.4 V, each for 5 s, while being exposed to various concentrations of thrombin.

**Figure 4:**
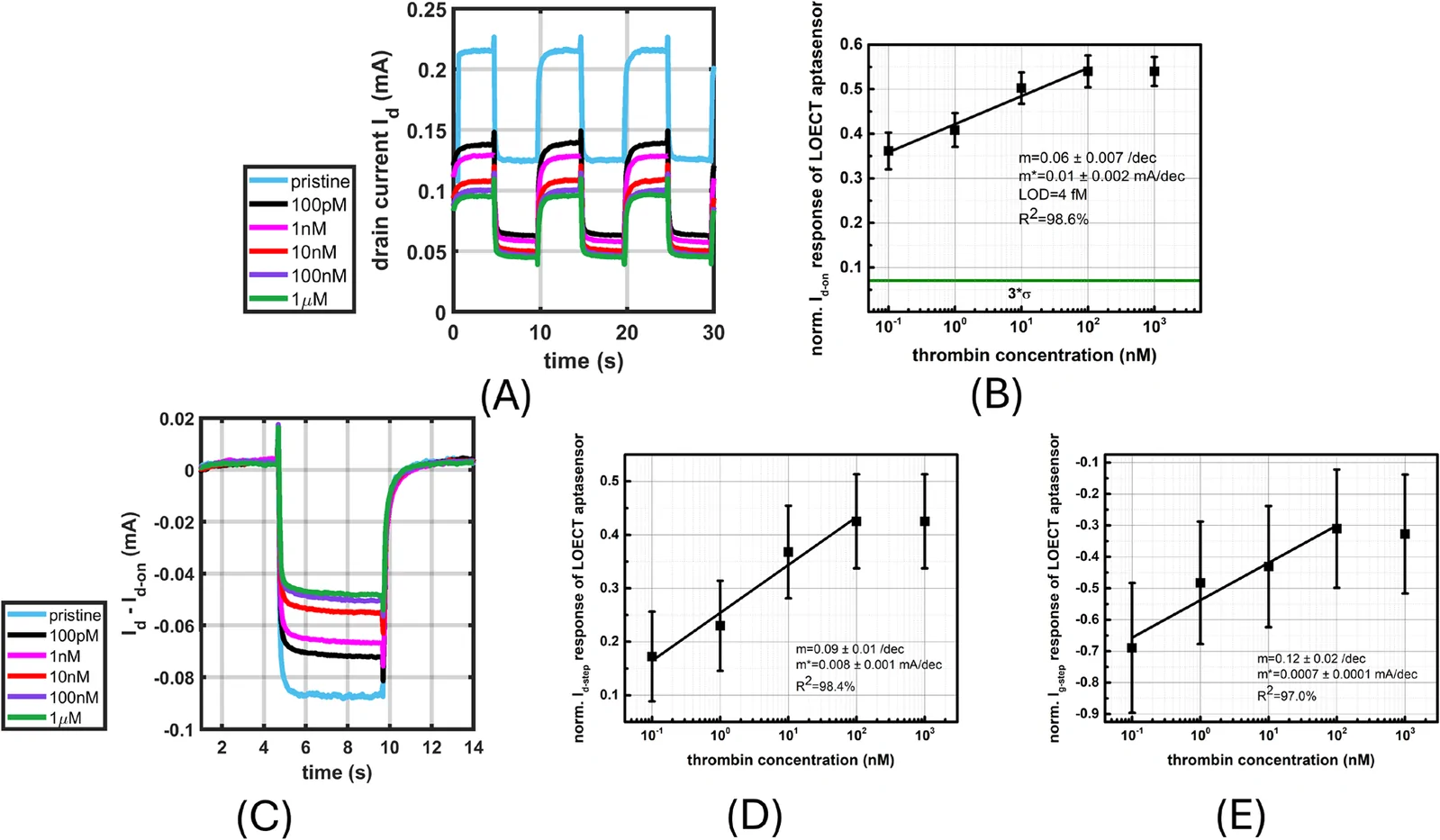
Response of LOECT-based aptasensors to thrombin using pulsed gate voltages *V*_g_. (A) Response of lateral OECTs with adsorbed TBA-29 and MCH (equal gate and channel area) to gate voltage pulses. The gate voltage *V*_g_ is pulsed between 0 and 0.4 V. The drain voltage *V*_d_ is kept constant at −0.2 V. The concentration of thrombin varies between 0 (pristine) and 1 μM. (B) Normalized response of the drain current *I*_d_ at *V*_g_ = 0 V from the pulsed measurement (cf. Figure 4A and Table S4), calculated using eq 3. (C) Temporal characteristic of adjusted drain current (*I*_d_-*I*_d‑on_) vs time, taken from (A), to observe a systematic decrease in the change in drain current with increasing concentration of thrombin. (D) Normalized difference of the *I*_d‑step_, calculated using eqs 4 and 5, and (E) *I*_g‑step_, calculated using eqs 6 and 7, calculated from (A) and Table S4 for drain current, and Figure S14 and Table S5 for gate current.

The change in drain current *I*_d_ for different thrombin concentrations is shown in Figure 4A, with the corresponding gate current *I*_g_ shown in Figure S14. The measured drain current *I*_d_ and gate current *I*_g_ at both gate voltages *V*_g_ = 0 (on state) and 0.4 V (off state) are presented as box-whisker plots (cf. Figure S15A–D) and histograms (cf. Figure S16A–D) with detailed statistical analysis (arithmetic mean and median) in Table S4 for the drain current and Table S5 for the gate current. In the following, the extracted values from the median statistical analysis are used.

Before thrombin exposure, the device exhibits a drain current *I*_d_ and gate current *I*_g_ of 213 μA and 2.5 μA at *V*_g_ = 0 V, respectively. Following a voltage pulse of 0.4 V, the OECT drain and gate currents change to 126 μA and 8.3 μA, respectively, corresponding to an ON/OFF ratio of 1.7 and amplification $A=\frac{\Delta I_{d}}{\Delta I_{g}}=15$ . This ON/OFF ratio is greater than that obtained from the measurement of the transfer characteristic of the LOECT with the same gate-to-channel ratio, or even greater than the ratio obtained for larger gates (gate-to-channel ratio of 10:1), shown in Figure 3A,C, respectively. This higher switching ratio indicates a deeper penetration of ions into the PEDOT:PSS channel as the gate voltage *V*_g_ is applied for a significantly longer duration for the pulsed *V*_g_ case than for the transfer characteristic.^60^

After exposure to thrombin, the on-drain current *I*_d‑on_ is reduced, as shown in Figure 4A. A similar change in *I*_d_ was previously observed in the transfer characteristic measurement shown in Figure 3A. A calibration curve is derived from the temporal characteristic of the OECT using eq 3, as shown in Figure 4B. The normalized and absolute sensitivity obtained for the device are 0.06/dec and 0.01 mA/dec, respectively. The LOD is determined to be 4 fM using eq 2, where the median absolute deviation of the drain current before thrombin exposure (cf. Table S4) is used as σ and $\frac{\Delta I}{I_{0}}$ random is set to 0.

In addition to the change in *I*_d‑on_, one can also observe a systematic change in the step size of the drain current *I*_d‑step_ defined by

4 $$I_{d‐\mathrm{step}}=I_{d‐\mathrm{on}}-I_{d‐\mathrm{off}}$$

This change can be correlated to a change in the effective gate voltage at the PEDOT:PSS-electrolyte interface. To observe this trend more clearly, each LOECT response is shifted by *I*_d‑on_, as shown in Figure 4C.

The normalized change in the drain current step, calculated by

5 $$\frac{\Delta I_{d‐step}}{I_{d‐\mathrm{step}‐0}}=\frac{I_{d‐\mathrm{step}}\left(pris\right)-I_{d‐\mathrm{step}}\left(thr\right)}{I_{d‐\mathrm{step}}\left(pris\right)}$$

is shown in Figure 4D, giving normalized and absolute sensitivity of 0.09/dec, and 0.008 mA/dec, respectively. This change in *I*_d‑step_ can be correlated to a change in the gate current (*I*_g‑step_) calculated as

6 $$I_{g‐\mathrm{step}}=I_{g‐\mathrm{off}}-I_{g‐\mathrm{on}}$$

7 $$\frac{\Delta I_{g‐\mathrm{step}}}{I_{g‐\mathrm{step}‐0}}=\frac{I_{g‐\mathrm{step}}\left(\mathrm{pris}\right)-I_{g‐\mathrm{step}}\left(\mathrm{thr}\right)}{I_{g‐\mathrm{step}}\left(\mathrm{pris}\right)}$$

Using eqs 6 and 7 and the temporal characteristic of the LOECT in Figure S14 and Table S5, a calibration curve is derived and shown in Figure 4E. Note that the normalized sensitivity *m* of the gate current (Figure 4E, 0.12 ± 0.02/dec) is almost identical to that of the drain current (Figure 4D, 0.09 ± 0.01/dec). However, the absolute sensitivity of the drain current (Figure 4D, 0.008 mA/dec) is increased by a factor of approximately 11 compared to the sensitivity of the gate current (Figure 4E, 0.0007 mA/dec), providing direct evidence of amplification of the electrochemical signal in an OECT aptasensor without influencing the normalized sensitivity.

### Detection of Thrombin with Vertical OECT Aptasensors

OECTs are usually fabricated in a lateral design, as presented in the previous sections. This design limits the downscaling of the channel length to approximately 1 μm due to limitations of photolithography techniques.^61^ However, as the transconductance of OECTs scales inversely with the channel length, further downscaling can result in increased transconductance and hence higher amplification.^47,49^

To scale the channel length beyond what is possible with standard lithography, OECTs were fabricated with vertically stacked source and drain electrodes, forming a vertical OECT (VOECT) as sketched in Figure 5A. Process details are summarized in the Experimental Section. In particular, PEDOT:PSS electropolymerization was used to grow PEDOT:PSS vertically between the source and drain electrodes with channel length, width, and thickness of 300 nm, 315 μm, and 300 nm, respectively.^47–49^ The area of the gate electrode is 0.15 mm^2^, resulting in a ratio between gate and channel area of ≈1500:1. The gate electrode of the VOECT is functionalized with TBA-29 or the random oligo aptamer and MCH, using the same procedure as for LOECTs. Considering the volume and volumetric capacitance of the electropolymerized PEDOT:PSS channel (28 μm^3^ and 150 F/cm^3^, cf. Figure S17A and Table S3), the capacitance of the electropolymerized PEDOT:PSS channel (*C*_ch_ = 4.2 nF) is comparable to the capacitance of the TBA-29/MCH-covered gate electrode (6 nF), resulting in a capacitance ratio *C*_g_/*C*_ch_ ≈ 1.4.

**Figure 5:**
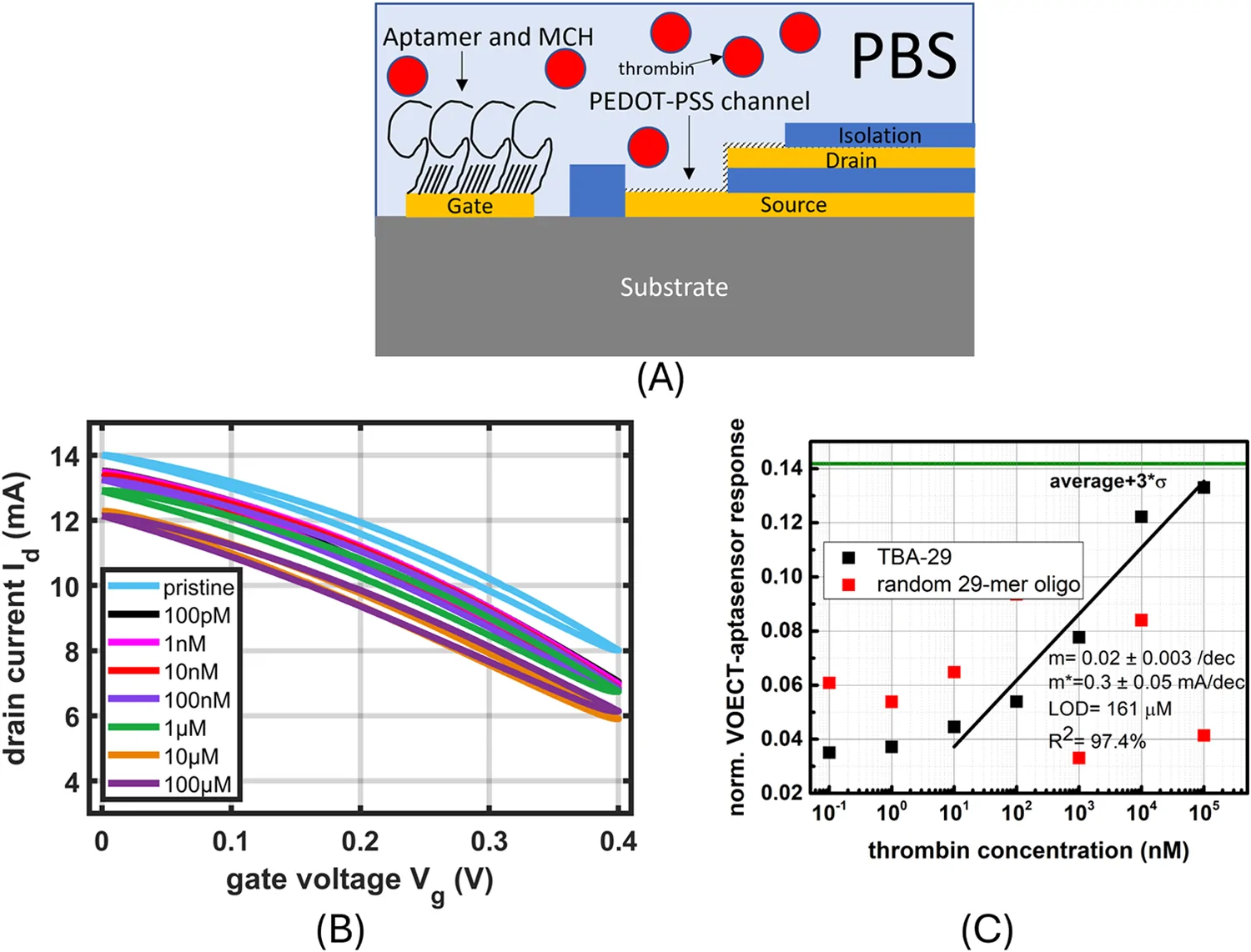
Response of VOECT-based aptasensors to thrombin using transfer characteristic measurements. (A) Illustration of a VOECT with stacked source and drain electrodes. A channel of PEDOT:PSS is grown electrochemically between a source and drain electrode. PEDOT:PSS channel and gate electrode are in contact with PBS 1.0× solution as both electrolyte and buffer solution for the thrombin analyte. TBA-29/random oligo and MCH molecules are deposited on the gate electrode. (B) Transfer characteristic *I*_d_ vs *V*_g_ of a vertical OECT (VOECT) with adsorbed TBA-29 and MCH on the gate for different thrombin concentrations. The gate electrode area and PEDOT:PSS channel width and length are 0.15 mm^2^, 315 μm, and 300 nm, respectively. (C) Normalized response of the VOECT-based aptasensor shown in Figure 5B. For comparison, the normalized response of random oligo-functionalized VOECT from Figure S17C is shown.

The transfer characteristic of a VOECT with adsorbed TBA-29 and MCH on its gate electrode before exposure to thrombin is shown in Figure 5B. The drain current reaches an on-current *I*_d‑on_ of 14 mA with an ON/OFF ratio of ≈1.8. The drain current and ON/OFF ratio of the VOECT are clearly higher than those of the LOECTs shown in Figures 3A,C and S13A, due to the shorter channel length and higher *C*_g_/*C*_ch_. After exposure to thrombin, the drain current of the VOECT decreases, similar to the behavior of LOECTs shown in Figure 3A,C. The corresponding gate current of the VOECT is shown in Figure S17B.

The correlation between the response of the VOECT-based aptasensor, calculated using eq 3, and the concentration of thrombin is shown in Figure 5C. A linear relationship is found with an absolute sensitivity m* of 0.3 mA/dec, which is the highest among the various aptasensor transducers in our experiments. However, this aptasensor is not sensitive to thrombin concentrations below 10 nM. Note that the normalized sensitivity of the aptasensor is significantly smaller than that of the other OECT-based sensors shown here, which already respond to concentrations of thrombin in the 100 pM range.

To understand the reason for the insensitivity of our VOECT-based aptasensor to low thrombin concentrations, the normalized response of the aptasensor is compared to the VOECT functionalized with the random-oligo (cf. Figure S17C,D for *I*_d_ vs *V*_g_ and *I*_g_ vs *V*_g_, respectively), shown in Figure 5C. Although the random-oligo-functionalized VOECT does not exhibit a linear relationship between its response and thrombin concentration, the response fluctuates at a magnitude comparable to the aptasensor response at 1 μM thrombin concentration. This fluctuating blank response of the VOECT-based aptasensor limits the sensor response at low concentrations and leads to a calculated LOD of 161 μM, which is higher than the highest thrombin concentration tested in our experiment.

Considering the lack of specific interaction between random 29-mer oligo and thrombin, the noise in the blank response of the VOECT-based aptasensor most likely does not originate from the gate. Instead, as the conductance of the OMIEC channel increases, the resistance of the metal interconnects and the contact between the source/drain electrode and the OMIEC channel become increasingly relevant, leading to higher noise in drain current *I*_d_.^62,63^ The role of metal interconnect resistance in limiting the performance of a VOECT has been confirmed by Skowrons et al.^47^ This resistance can be eliminated by using four-point probe measurements.

Moreover, PEDOT:PSS has been shown to be sensitive to thermal treatment, which, in combination with the presence of oxygen and water in the environment, leads to morphological and chemical changes in the material, causing fluctuations in the OMIEC conductivity.^64,65^ The drain current *I*_d_ in our VOECT-based aptasensor is much higher than in lateral OECTs, which might cause local heating in the channel, inducing fluctuations in its conductivity. This mechanism of conductivity fluctuation can be averted by applying a lower drain voltage *V*_d_ in the measurement, which is also advantageous to reduce the operation voltage of the device and to prevent voltage-induced desorption of the molecules from the gate.^20–22^

In summary, the use of a vertical OECT architecture as a transducer for aptasensors is demonstrated for the first time in this work. As expected, a VOECT-based aptasensor exhibits an increase in drain current and, hence, an increase in absolute sensitivity of the devices m*, surpassing the LOECT-based sensor in the current experiments. Note that the absolute sensitivity of our VOECT-based aptasensor is higher than that of the majority of reported OECT-based aptasensors.^16,29,33,34,56,57^ The VOECT-based aptasensor shows comparable, or even higher, sensitivity than the work of Bidinger et al. and Ji et al., which both use a redox reporter tag and pulsed variation of OECT gate voltage.^16,34^ On the other hand, the absolute sensitivity of our VOECT-based aptasensor is lower than the work of Peruzzi et al. for thrombin detection, despite a narrower linear detection range for the aforementioned work compared to our OECT-based aptasensor.^33^ Nevertheless, in the experiments shown here, this improvement comes at the expense of additional noise in control experiments, significantly increasing the calculated LOD.

### Comparison of the Different Designs

Although all sensor designs are sensitive to thrombin, their performance varies strongly. The normalized and absolute sensitivity of the aptasensors are compared in Figure 6A,B, respectively. Overall, three terminal, OECT-based devices show a clear increase in absolute sensitivity over standard CV or DPV measurements. Furthermore, optimization of the area of gate and PEDOT:PSS channel is crucial to optimize the sensitivity. A maximum of normalized sensitivity is reached for a gate area equal to the channel (500 μm × 500 μm) and drops for both smaller and larger gate electrodes. However, one has to keep in mind that the absolute sensitivity might differ. Here, the vertical OECT shows the highest absolute sensitivity (cf. Figure 6B).

**Figure 6:**
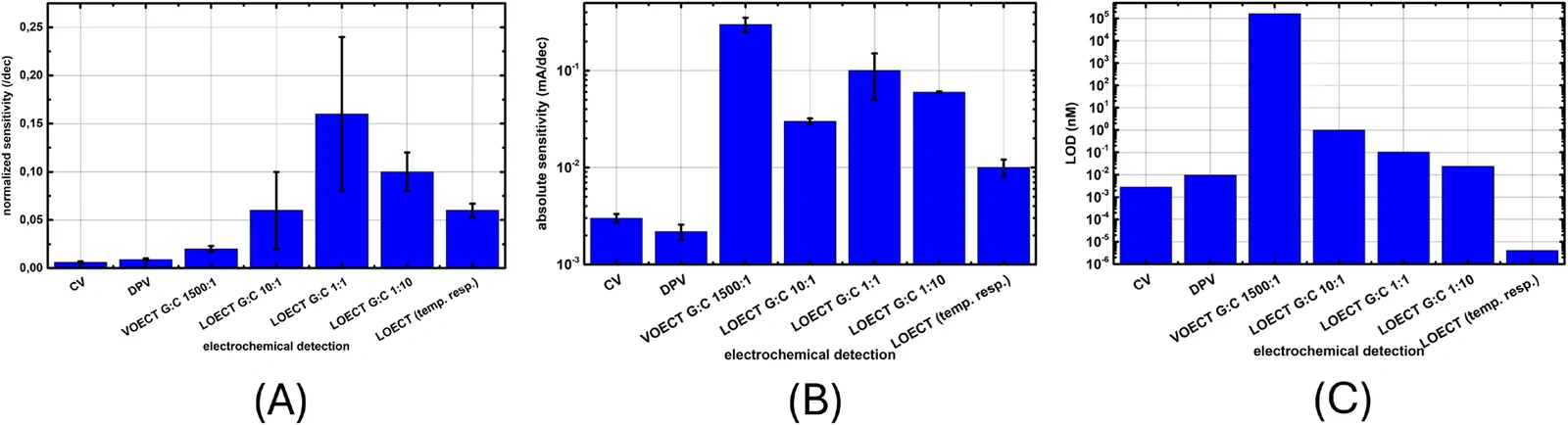
Comparison of normalized sensitivity, absolute sensitivity, and the limit of detection of the different aptasensors. (A, B) OECT-based sensors show a clear advantage over standard CV- and DPV-based sensors. An optimized (A) normalized sensitivity is found for LOECT with an equal area of gate and channel. (B) VOECT-based sensor shows the highest absolute sensitivity among the tested thrombin aptasensors. (C) Two strategies for optimization of the LOD can be identified: reduction of gate area and using pulsed measurements.

Aptasensor sensitivity is not the only benchmark for thrombin sensors, but the LOD is almost equally important. A comparison of the LOD of the different designs is shown in Figure 6c. Two strategies can be identified to reduce LOD. First of all, a reduction in the gate area below the area of the channel decreases the LOD. Second, changing the measurement protocol to a pulsed gate voltage results in the lowest LOD.

The trend of optimized normalized sensitivity, as shown in Figure 6A, can be partially explained as a consequence of fewer aptamers contributing to the total ionic signal.^17^ However, as the number of adsorbed aptamers decreases, the importance of molecular desorption increases as well and can dominate the response of an aptasensor due to analyte binding. This can explain the apparent drop in normalized sensitivity, as the area of aptamer-adsorbed gate decreases from the gate-to-channel area ratio 1:1 to 1:10.

### Stability and Selectivity

Aside from a high sensitivity and satisfactory LOD, long-term stability and selectivity are essential for a realistic implementation of the aptasensor.^18,20–22^ Other than the LOECT with gate-to-channel area ratio 1:10, there was no indication of voltage-induced desorption of the aptamers for both the OECT-based sensors tested using scanned or pulsed gate voltage *V*_g_, over the whole course of measurements (3–4 h). However, the aptamers might desorb from the gate at a relatively lower rate, which is not visible in the total duration of experiments and would only be relevant after extended testing.^20–22^

To test the selectivity of aptasensors, one OECT with a gate-to-channel area ratio of 1:1 and TBA-29/MCH functionalization is exposed consecutively to BSA, insulin, and thrombin, each for 30 min incubation with a washing step in between, as shown in Figure S16A,B. Overall, the aptasensor showed excellent selectivity to thrombin. However, we do not expect our aptasensor to resist prolonged exposure beyond 10 h to blood’s cellular components or ruptured cells.^23^ The imperfection in substrate protection, as provided by the MCH SAM, has been shown to lead to molecular desorption or biofouling.^20–23^ A more reliable protection of an aptasensor can be achieved by using an agarose membrane or 8-mercapto-1-octanol (MCO) SAM passivation.

### Discussion

The response of the OECT transducer to aptamer interaction with analytes on the gate cannot be fully described using the Bernards and Malliaras model, which only evaluates the modulation of drain current *I*_d_. To account for the change in *I*_d_ at 0 V (*I*_d‑on_), which presumably is caused by the charges at the backbone of adsorbed aptamers on the gate, the Bernards and Malliaras model can be modified as follows^36,37,54^

8 $$V_{\mathrm{ch}}=V_{g}\frac{1}{1+\frac{C_{\mathrm{ch}}}{C_{g}}}+V_{\mathrm{sp}}\left(A_{1},A_{2},A_{3},...A_{N}\right)e^{-d/D}$$

where *V*_ch_ is the potential difference between the channel and electrolyte, *V*_g_ is the potential difference between the gate and channel, *C*_ch_ is the capacitance at the interface between the channel and electrolyte, *C*_g_ is the capacitance at the interface between the gate and electrolyte, and *V*_sp_ represents the surface potential due to the electrostatic interaction between the electrolyte and the negative charges at aptamer backbones, which depends on the different conformations of the aptamer (*A*_N_) on the gate. On the other hand, *d* represents the distance between aptamer backbones and the surface of gate electrode, while *D* represents the Debye length, which is typically between 0.7 and 1 nm in PBS buffer.^66^

Note that the ratio between *C*_g_ and *C*_ch_ in our experiments is considered to be low. Therefore, the first term of eq 8 shifts the channel potential only weakly, resulting in a low ON/OFF ratio when scanning the gate voltage in the transfer characteristic. Still, the second term in eq 8 leads to a significant response to thrombin. The shift in channel potential with gate voltage *V*_g_ can be increased by using pulsed gate voltages because more time is given for the OMIEC channel to respond, leading to a higher ON/OFF ratio as observed here.

## Conclusion

Thrombin is a crucial enzyme for the wound healing process, reaching levels of μM at the site of injury during a coagulation process. An abnormal level can also indicate several diseases, such as COVID-19 and leukemia. Often, thrombin is detected by aptamer bioreceptors in a standard electrochemical setup, providing a reasonable sensitivity. However, the sensor signal, i.e., a change in current, scales with the electrode area, reducing the signal-to-noise ratio and hindering sensor miniaturization.

To increase the signal strength of thrombin aptasensors, a standard passive aptasensor based on TBA-29 is successfully combined with lateral and vertical OECTs, leading to an increase in the absolute sensitivity. It is shown that the sensitivity and detection range of our OECT-based aptasensor can be adjusted by the area of the gate electrode and the active channel. OECTs with a gate-to-channel area ratio of 1:1 appear to exhibit the highest normalized sensitivity, while VOECTs show the highest absolute sensitivity.

Aside from sensitivity, it is shown that the limit of detection (LOD) of the aptasensors can be minimized by either decreasing the gate area or by using a different interrogation protocol, viz., applying gate voltage pulses instead of full gate voltage sweeps.

Overall, the OECT-based sensors shown here result in a significant increase in performance, even at a gate voltage *V*_g_ = 0 V. This reduction in operation voltage is significant since it allows the sensor to operate in a very low voltage range, thus avoiding any unwanted electrochemical processes at the gold surface. The trends identified here are important design rules to optimize OECT aptasensors beyond thrombin, moving this sensor design further toward an application in clinical settings.

## Supplementary Material



## Notes

Electrochemical interrogation of TBA-29/random 29-mer oligo and MCH binary SAM in a classical setup using DPV, CV, and EIS; analysis of EIS data; output curves of LOECT; transfer characteristic of LOECTs with adsorbed TBA-29/random 29-mer oligo and MCH on the gate; temporal characteristic analysis of the LOECT-based aptasensors with gate-to-channel area ratio 1:1; transfer characteristic of the VOECT with adsorbed TBA-29/random 29-mer oligo and MCH on the gate; selectivity test of an LOECT-based aptasensor for thrombin; and comparison of different electrochemical methods for thrombin detection in the literature (PDF)
