# Supplementary material for: Miniaturization of Aptasensors in Organic Electrochemical Transistors for Sensitivity Enhancement
Source: Anal Chem. 2026 Aug 21;98(35):25646–58. doi: 10.1021/acs.analchem.6c02236 (PMC13564037; doi:10.1021/acs.analchem.6c02236)
Supplement: Supplementary file 1 [file ac6c02236_si_001.pdf]

Supporting Information: Miniaturization of Aptasensors in Organic Electrochemical Transistors for Sensitivity Enhancement

Andika Asyuda,[*,†] Rasheed T. Odunbaku,[†] Luka J.L. Striethorst,[†] Henrique F.P. Barbosa,[†] Andreas Schander,[†] Ramesh Kumar,[†,‡,¶] Dur E.N. Gakhar,[†] Sarah Bornemann,[†] Rachel E. Armstrong,[§,‖] and Björn Lüssem[*,†,⊥]

†*Institut für Mikrosensoren, -Aktoren, und -Systeme (IMSAS), Universität Bremen, Bremen, 28359, Germany*

‡*Light Technology Institute, Karlsruhe Institute of Technology, Karlsruhe, 76131, Germany*

¶*InnovationLab GmbH, Heidelberg, 69115, Germany*

§*OnePlanet Research Center, Wageningen, 6708 WH, the Netherlands*

‖*imec, Wageningen, 6708 WH, the Netherlands*

⊥*MAPEX Center for Materials and Processes, Universität Bremen, Bremen, 28334, Germany*

E-mail: asyuda@uni-bremen.de; bluessem@uni-bremen.de

Contents:

1. Pages: 16
2. Figures: 18
3. Tables: 5

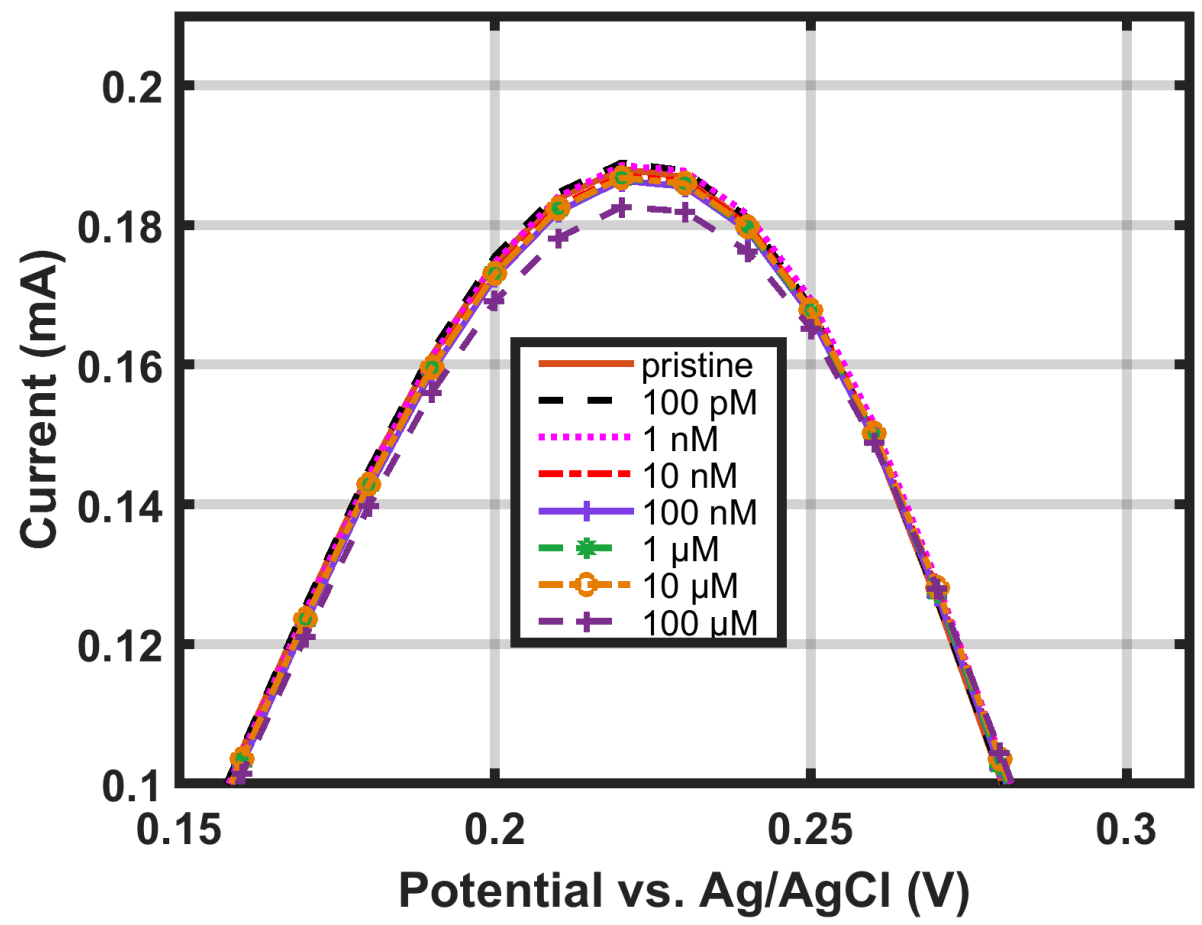

Figure S1: **Random 29-mer oligo interrogation using DPV**. The measurement was performed in a standard electrochemical cell, consisting of three electrodes, while the sample was exposed to increasing concentrations of thrombin, varying between 100 pM and 100 μM.

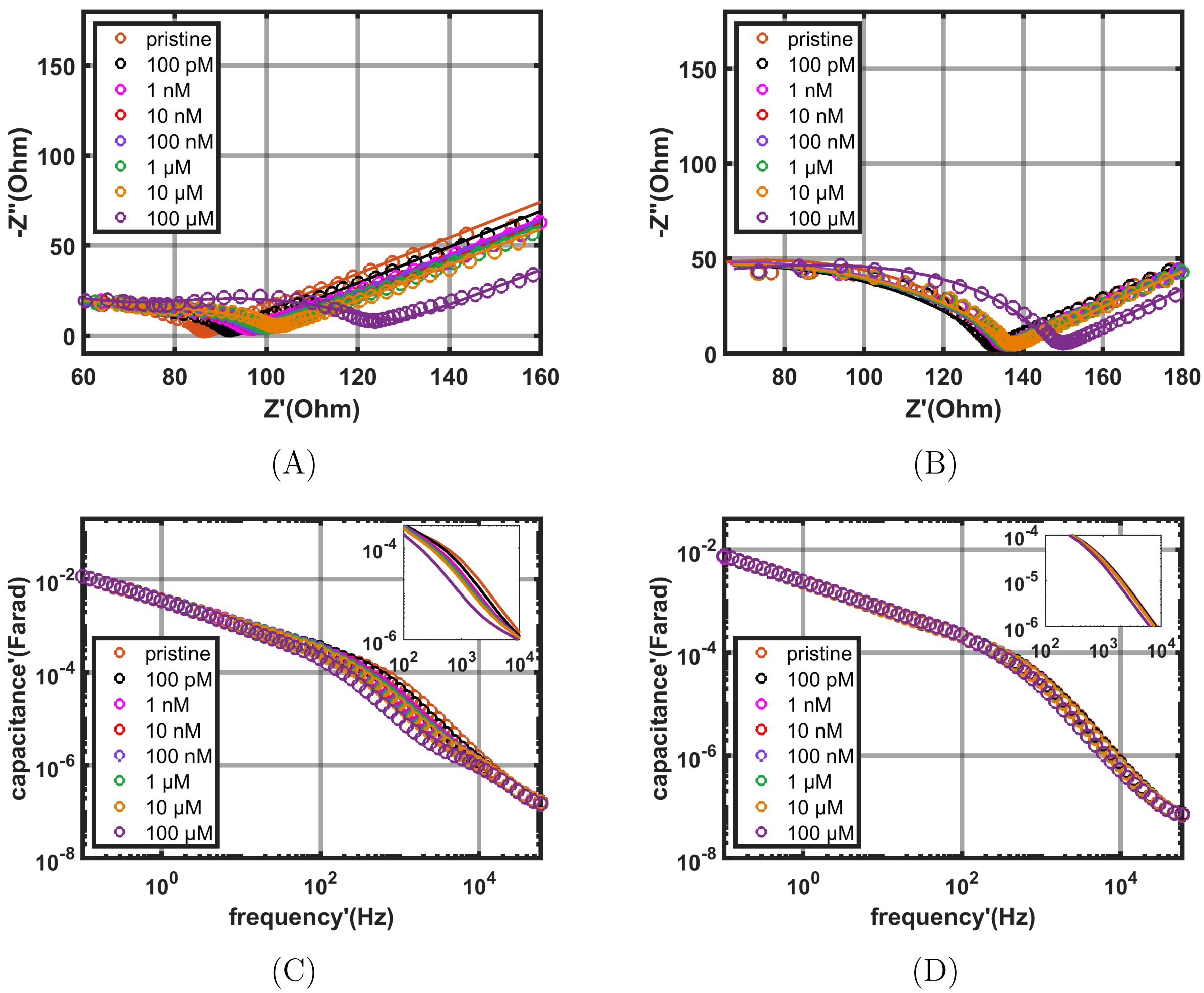

Figure S2: **Aptasensor interrogation using EIS**. The spectra of (**A**) TBA-29 and (**B**) random 29-mer oligo SAM was taken in a three-electrode electrochemical cell, as described in Experimental Section. The samples were exposed to different concentration of thrombin between 100 pM and 100 μM. The spectra of TBA-29 exhibits a systematic shift in the real impedance, which the transition between charge transport and mass transport takes place in the system. For the random oligo, this shift is only observed at thrombin concentration 100 μM, presumably due to biofouling of the sample by a high concentration of thrombin. (**C**) The TBA-29 also exhibits a decrease in total capacitance of the system with increasing thrombin concentration, in contrast with (**D**) the random oligo.

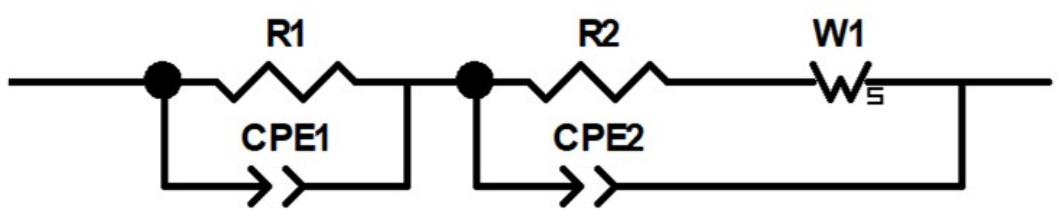

(A)

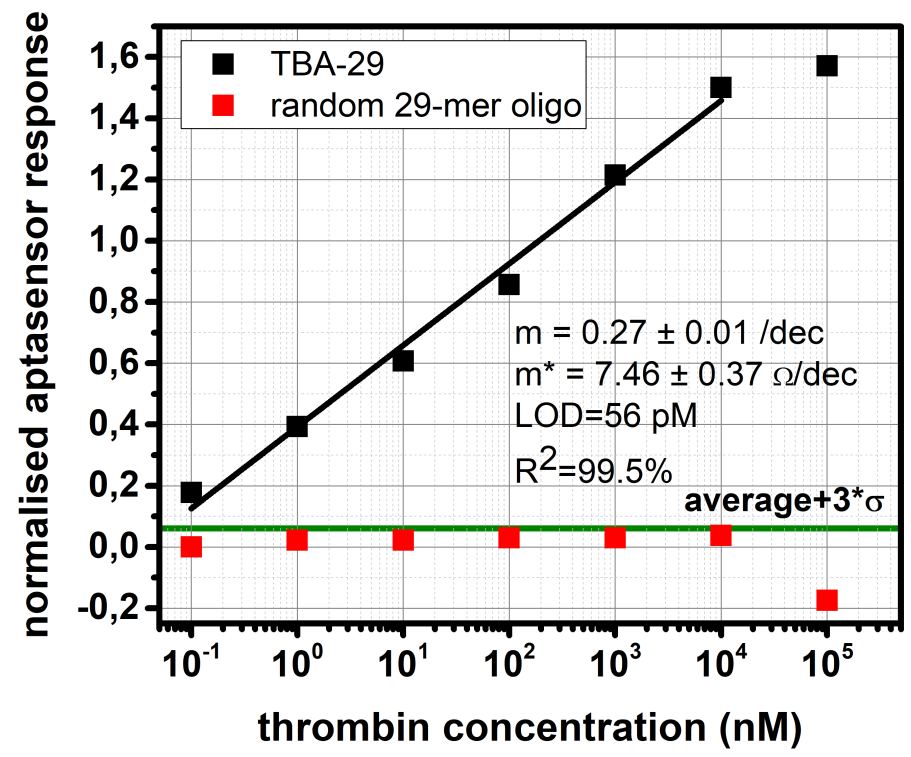

(B)

Figure S3: **Modified Randles circuit and calibration curve for the EIS interrogation of thrombin aptasensor**. (**A**) Modified Randles equivalent circuit to model the EIS in the Figures S2**A** and **B**. R1 and CPE1 represents the geometrical resistance and constant phase element, respectively, while R2, W1, and CPE2 represents the interfacial resistance, Warburg element, and constant phase element, respectively. (**B**) The normalized change of interfacial resistance R2 in Table S1 for TBA-29 aptasensor and random oligo, defined as the following: $\frac{\Delta I}{I_0} = \frac{R_{thr} - R_{pris}}{R_{pris}}$ The aptasensor LOD, normalized-, and absolute sensitivity are 53 pM, 0.27 /dec, and 7.5 Ω/dec, respectively.

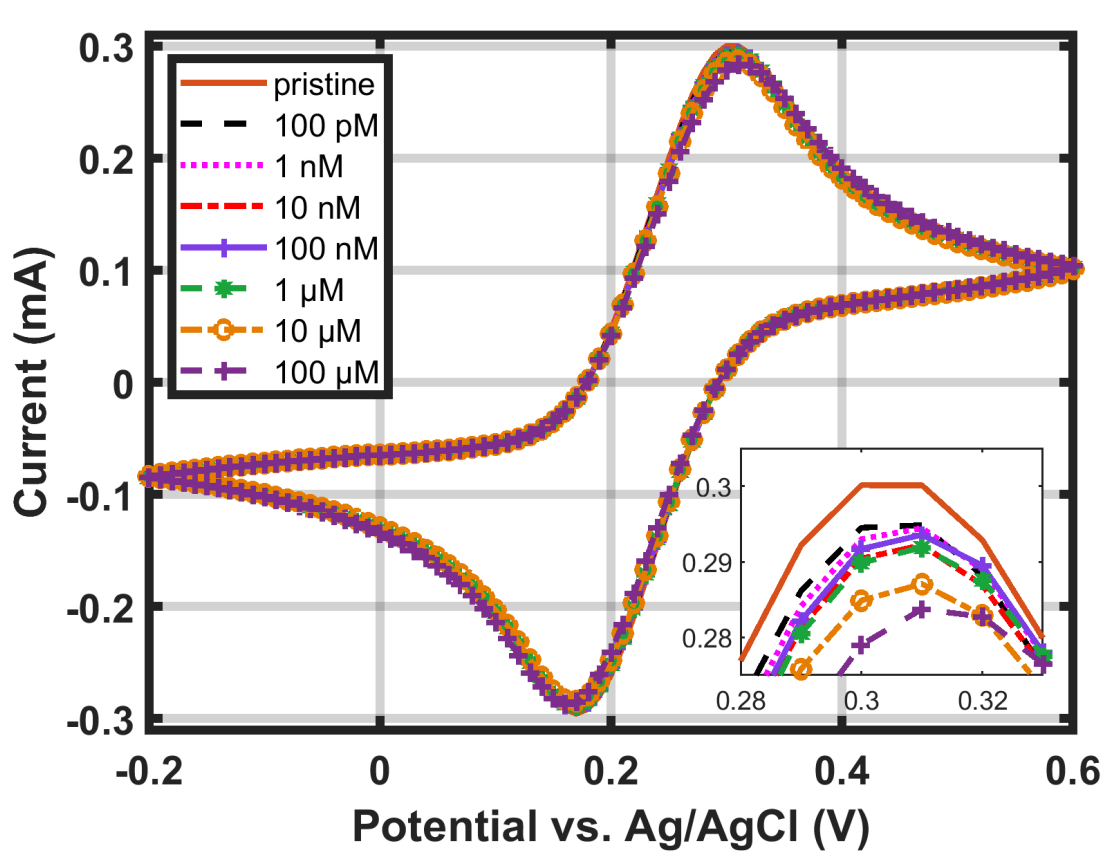

(A)

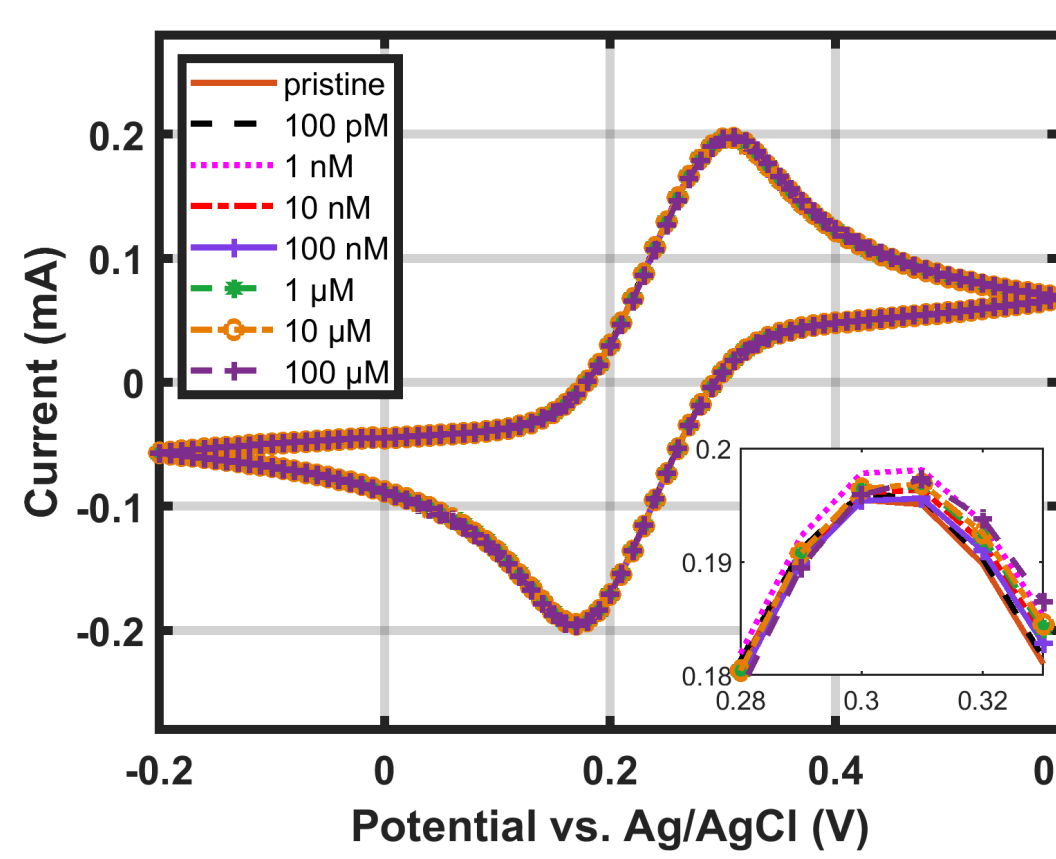

(B)

Figure S4: **Aptasensor interrogation using CV**. The measurements of (**A**) TBA-29 and (**B**) random 29-mer oligo SAM on gold substrate were performed in a standard electrochemical cell consisting of three electrodes, while the samples were exposed to different concentration of thrombin between 100 pM and 100 μM. The oxidation peak around 0.3 V is magnified in inset for clarity.

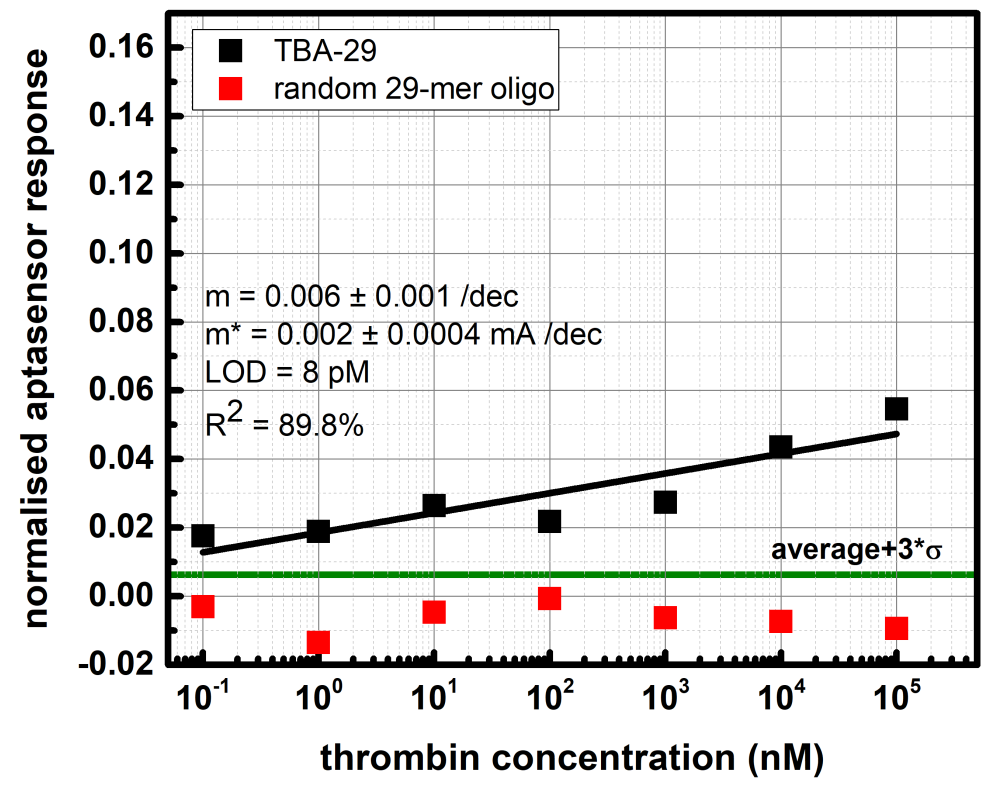

Figure S5: **The calibration curve for the CV interrogation of thrombin aptasensor**. The normalized response from CV measurement of TBA-29 sensor (cf. Figure S4**A**) and random 29-mer oligo (cf. Figure S4**B**) are calculated using the current of CV oxidation peak and Equation 1. The aptasensor LOD, normalized-, and absolute sensitivity are 8 pM, 0.006 /dec, and 0.002 mA/dec, respectively.

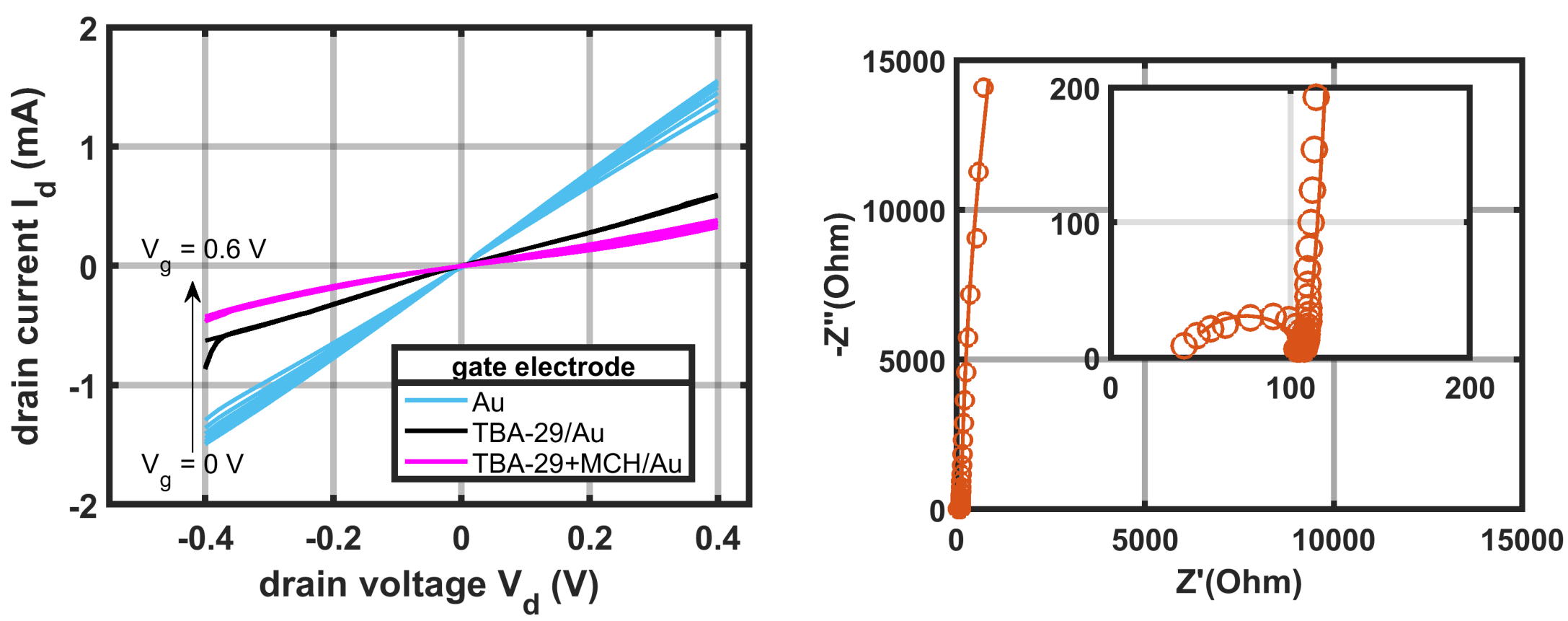

(A) (B)

Figure S6: **Output curves of LOECT and EIS of spin-coated PEDOT:PSS channel**. (**A**) Output curve I_d vs V_d, measured at increasing V_g between 0 and 0.6 V, for the LOECTs with equal area of gate and channel (0.25 mm^2), with clean gold gate electrodes, after the adsorption of TBA-29, and subsequently MCH on the gate. The ranges of V_g and V_d sweep are kept relatively low to prevent unwanted electrochemical reaction on the gate. (**B**) EIS of spincoated PEDOT:PSS with volume $2*10^{-3}$ mm^3, showing a semicircle at high frequency (cf. inset), suggesting a charge transport, and Warburg process at low frequency. The spectrum is fitted with an equivalent circuit (cf. Figure S7**A**) and presented in table S3.

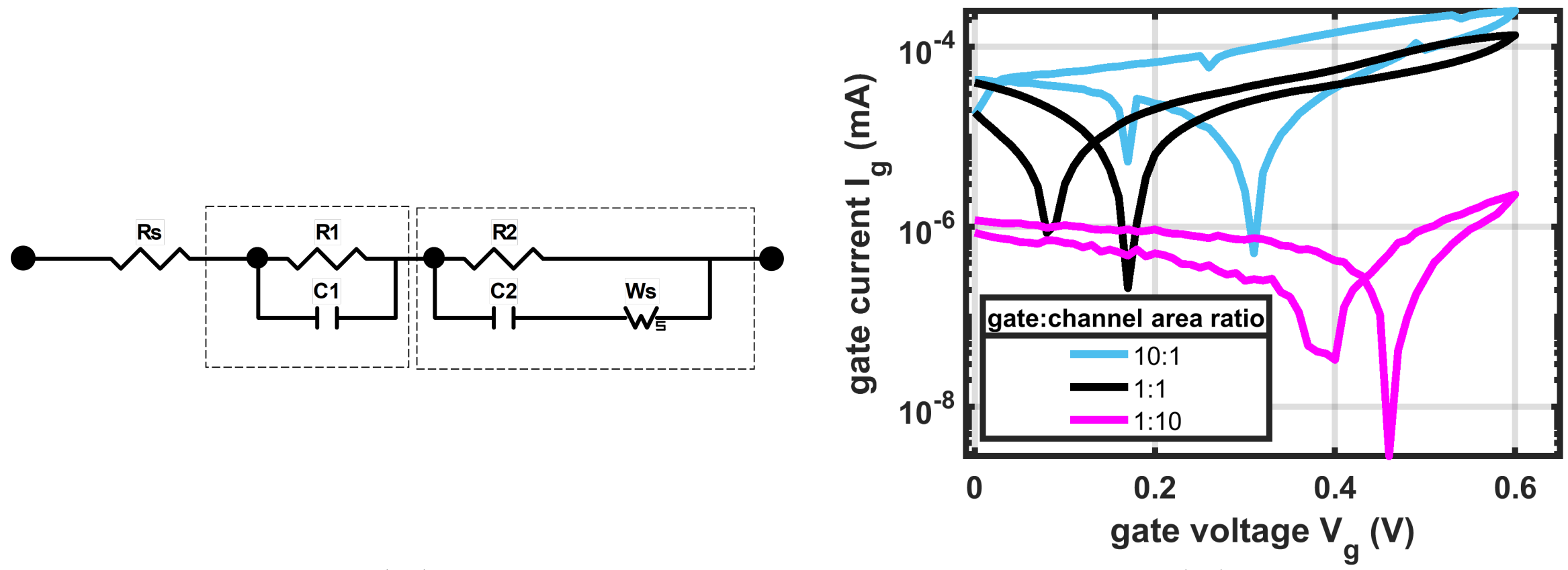

(A) (B)

Figure S7: **Modifed Randles circuit and I_g vs V_g of LOECT-based aptasensor.** (**A**) An equivalent circuit to fit EIS of PEDOT:PSS channel. The parameter Rs, R1, C1, R2, C2, and Ws describe electrolyte resistance, charge transport resistance, geometrical capacitance, interfacial resistance, double layer capacitance, and Warburg, respectively. (**B**) Transfer characteristic I_g vs V_g for the LOECTs with different area ratio between the gate and the channel: 10:1, 1:1, and 1:10. V_d is kept constant at -0.2 V during the measurement.

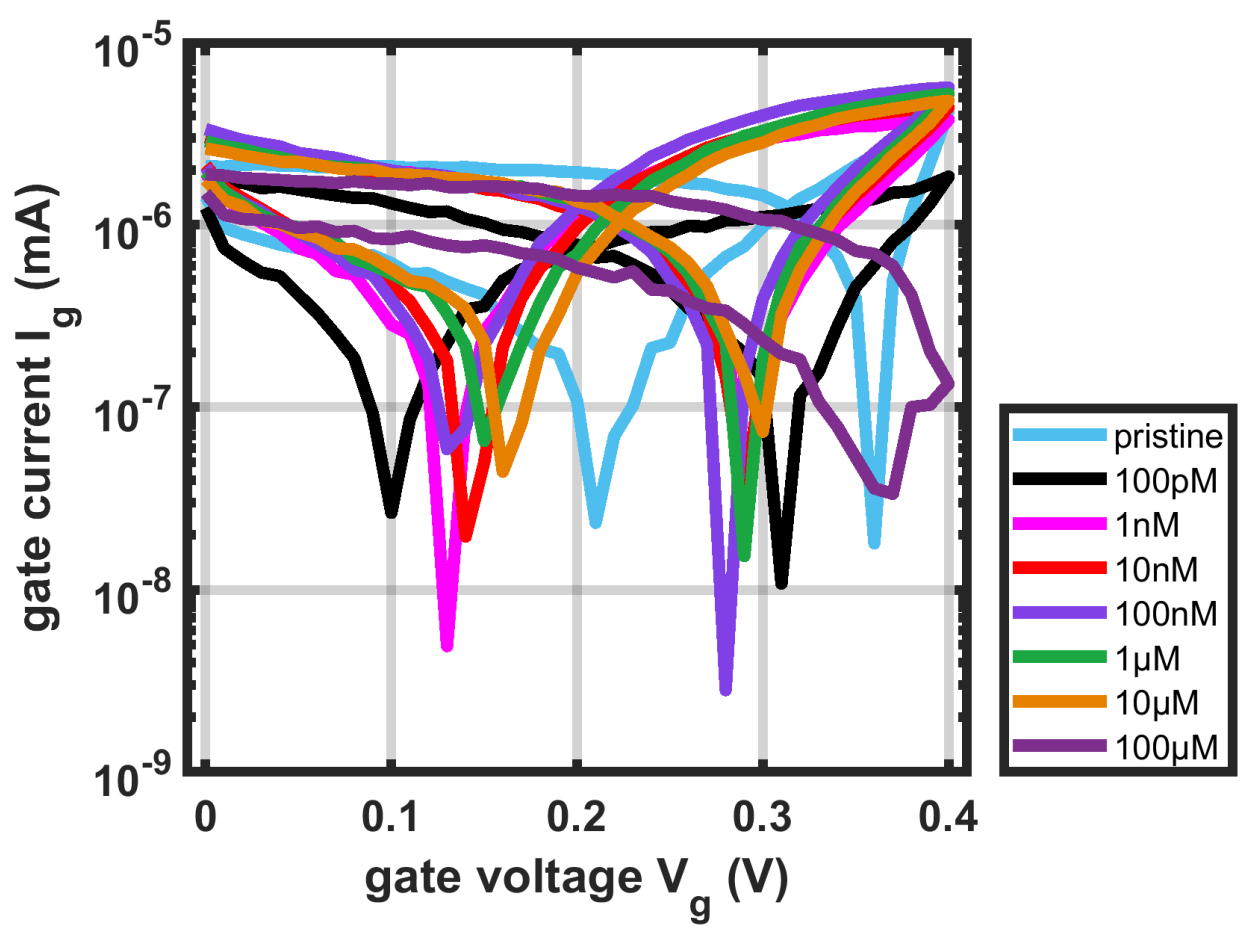

Figure S8: **Interrogation of the LOECT with adsorbed TBA-29 and MCH on the gate (gate to channel area ratio 1:1) with I_g vs V_g.** During the interrogation, the sample is exposed to different concentration of thrombin in the electrolyte. The drain voltage V_d is kept constant at -0.2 V.

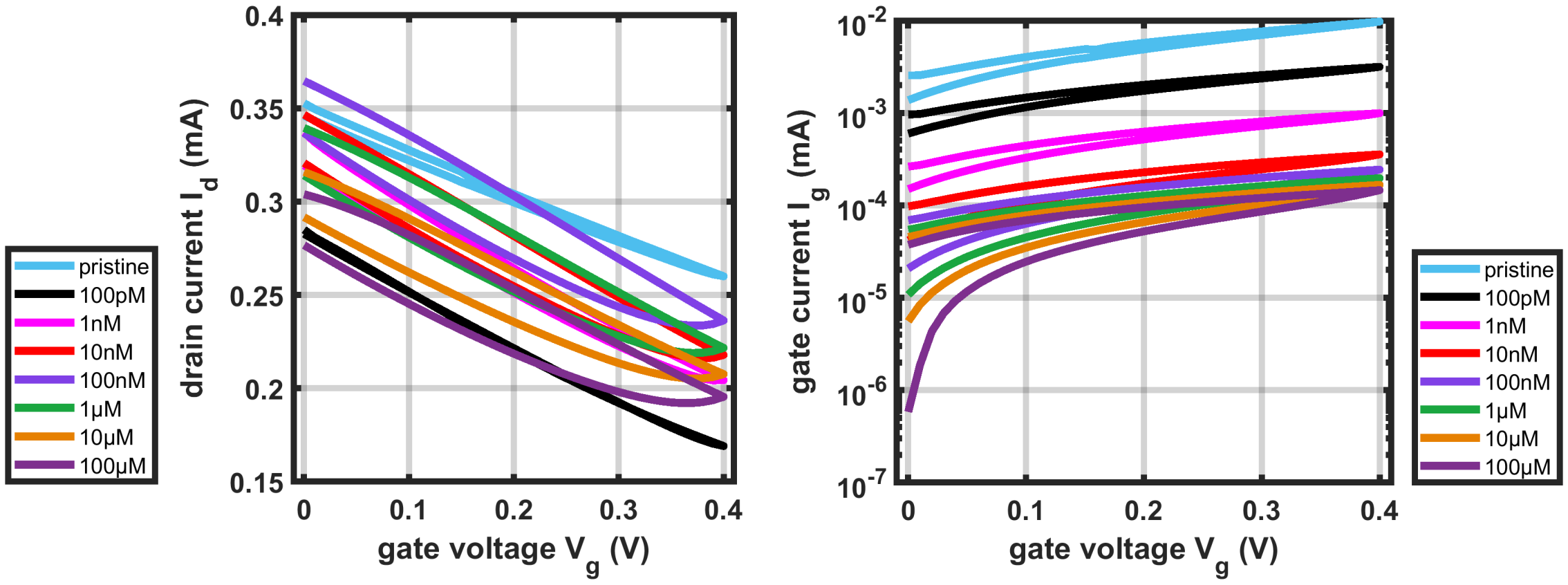

(A) (B)

Figure S9: **Interrogation of the LOECT with adsorbed random-29-mer oligo and MCH on the gate (gate to channel area ratio 1:1) with transfer characteristic**. The relation between (**A**) I_{d} vs V_{g} and (**B**) I_{g} vs V_{g} are shown at different concentration of thrombin exposure in the electrolyte. The drain voltage V_{d} is kept constant at -0.2 V.

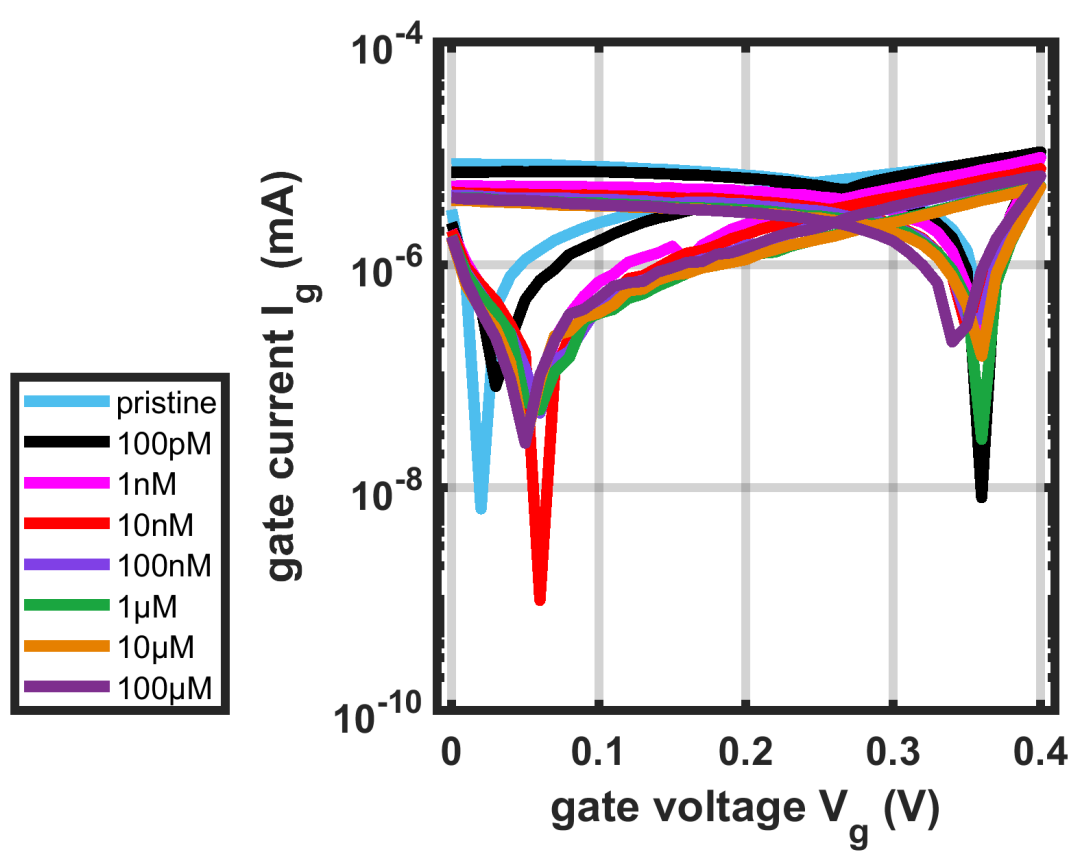

Figure S10: **Interrogation of the LOECT with adsorbed TBA-29 and MCH on the gate (gate to channel area ratio 10:1) with I_{g} vs V_{g}**. During the interrogation, the sample is exposed to different concentration of thrombin in the electrolyte. The drain voltage V_{d} is kept constant at -0.2 V.

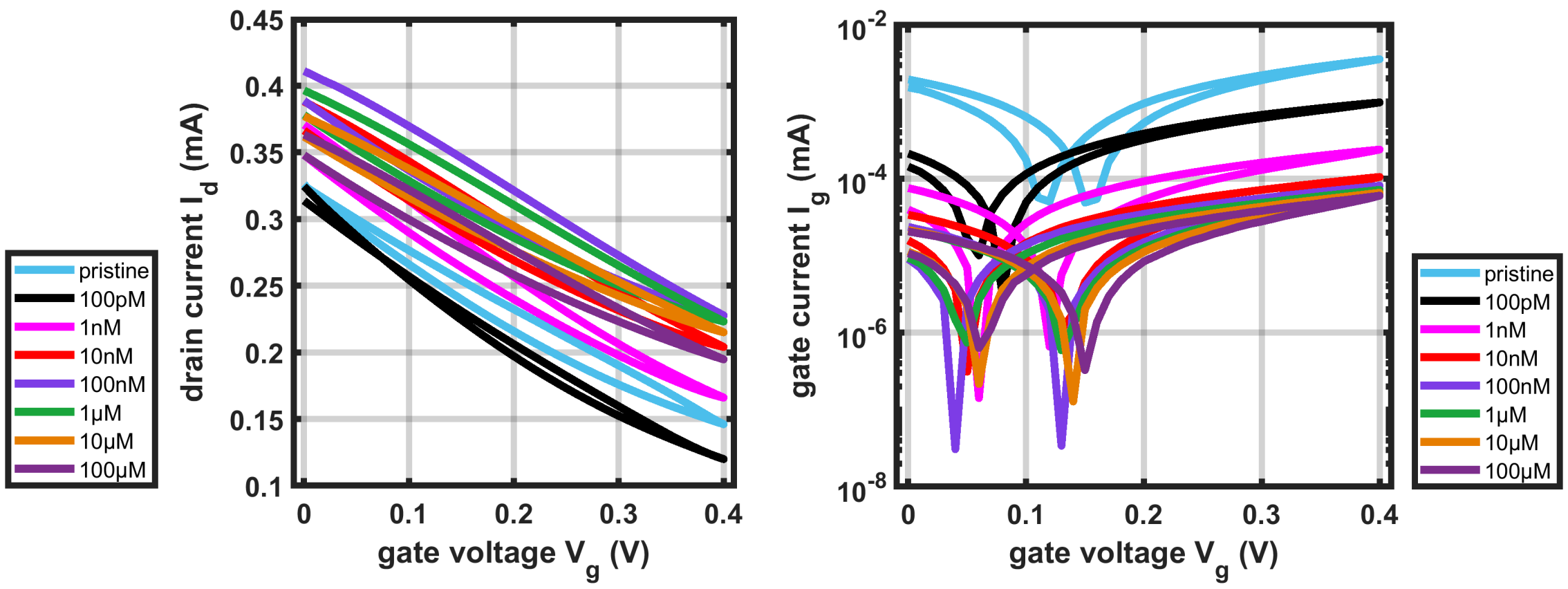

(A) (B)

Figure S11: **Interrogation of the LOECT with adsorbed random-29-mer oligo and MCH on the gate (gate to channel area ratio 10:1) with transfer characteristic**. The relation between (**A**) I_d vs V_g and (**B**) I_g vs V_g are shown at different concentration of thrombin exposure in the electrolyte. The drain voltage V_d is kept constant at -0.2 V.

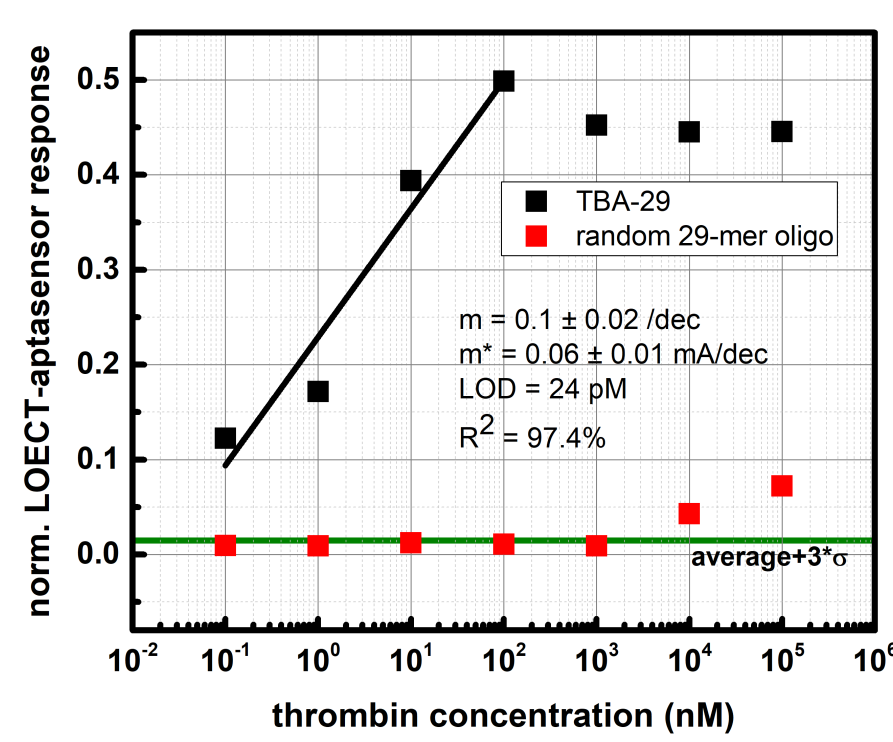

Figure S12: **Calibration curve of LOECT-based aptasensors with gate to channel area ratio of 1:10 to varying thrombine concentrations**. A consistent response is shown similarly to Figure 3**B** and **D**, with m and m* 0.1 /dec and 0.06 mA/dec, respectively. The normalized response of LOECT with adsorbed TBA-29 (from Figure S13**A**) and random 29-mer oligo (from Figure S13**C**) on the gate are calculated using Equation 3.

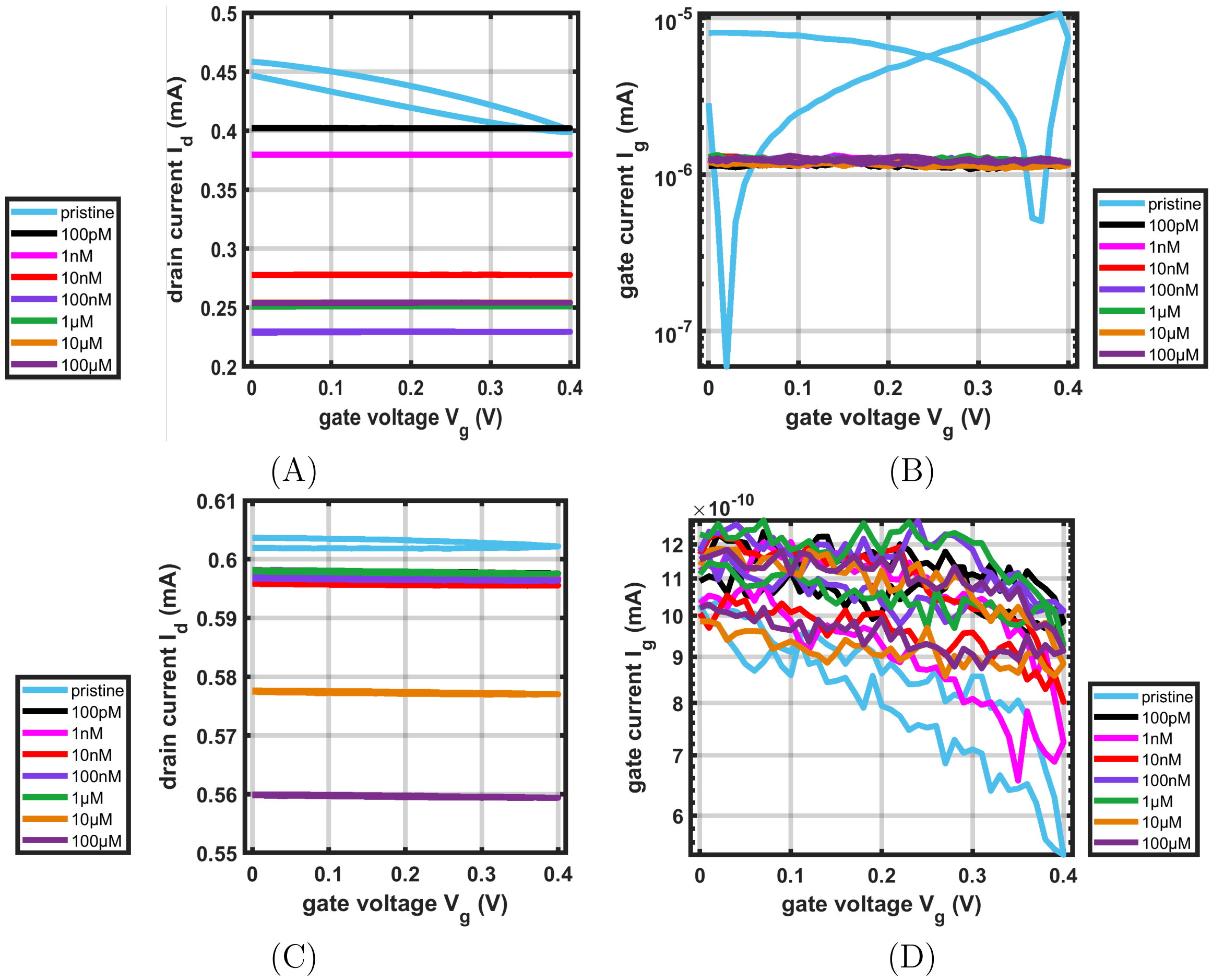

Figure S13: **Interrogation of the LOECT with adsorbed DNA oligo and MCH on the gate (gate to channel area ratio 1:10) with transfer characteristic**. The gates are functionalised with MCH and either (**A** and **B**) TBA-29 or (**C** and **D**) random 29-mer oligo. The relation between (**A** and **C**) I_d vs V_g and (**B** and **D**) I_g vs V_g are shown at different concentration of thrombin exposure in electrolyte. The drain voltage V_d is kept constant at -0.2 V

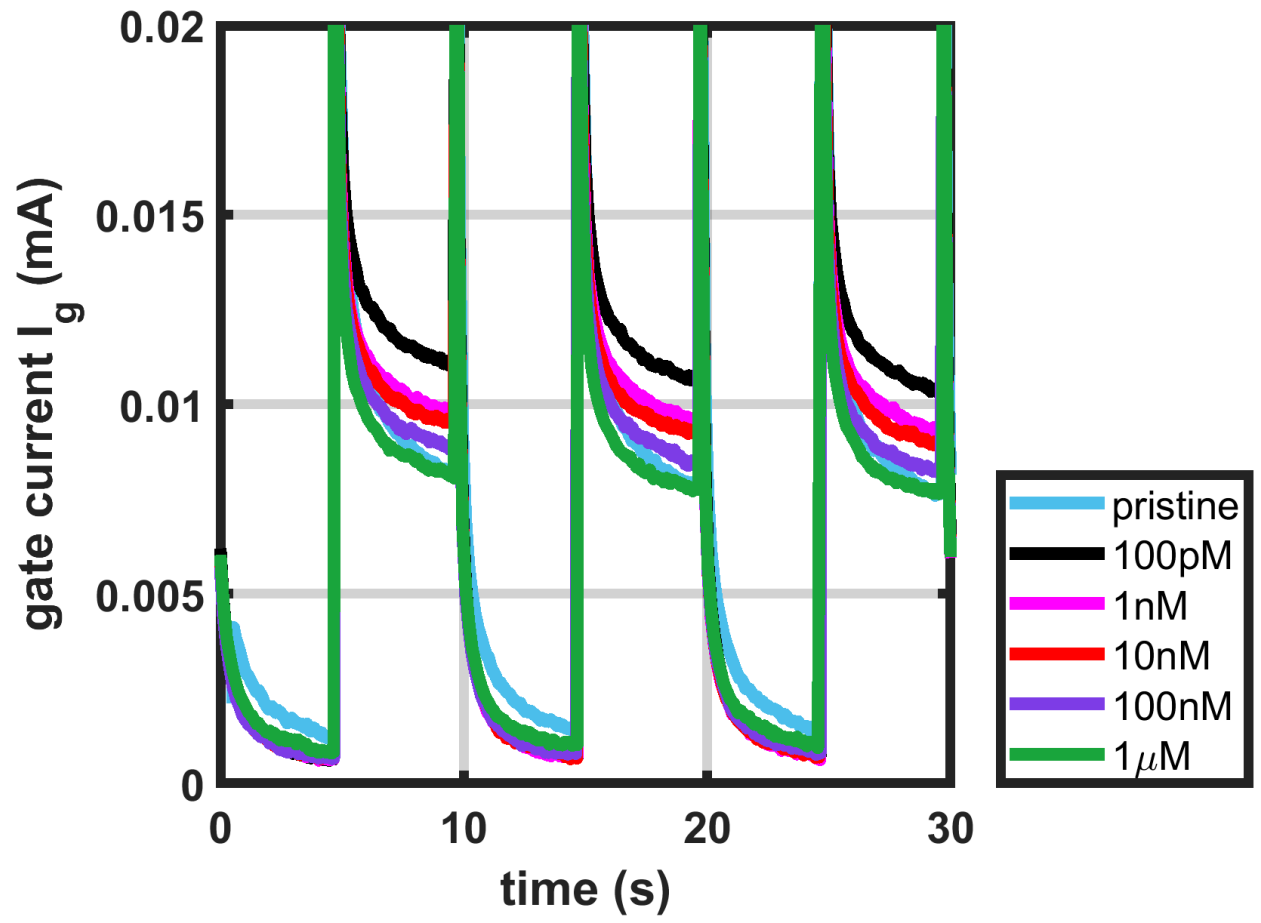

Figure S14: **Temporal characteristic gate current I_g vs time of a LOECT with adsorbed TBA-29 and MCH on gate electrode and gate:channel area ratio 1:1**. The gate voltage V_g is pulsed between 0 V (0 s≤t≤5 s, 10 s≤t≤15 s, and 20 s≤t≤25 s) and 0.4 V (5 s≤t≤10 s, 15 s≤t≤20 s, and 25 s≤t≤30 s), each for 5 s. The drain voltage V_d is kept constant at -0.2 V. The concentration of thrombin in electrolyte varies between 0 (pristine) and 1 μM.

Table S1: **Electrochemical circuit analysis for TBA-29 and random-29mer oligo SAM**. Parameter of electrochemical circuit in Figure S3**A** to model the EIS data in Figure S2**A** and S2**B**, for TBA-29 and random 29-mer oligo SAM sample on gold, respectively.

thrombin exposure	aptamer/random	R1 (Ω)	C1 $\left(\frac{s^{N1}}{Ohm}\right)$	N1	R2 (Ω)	W1 $\left(\frac{\sqrt{s}}{\Omega}\right)$	C2 $\left(\frac{s^{N2}}{Ohm}\right)$	N2
pristine	aptamer	58	$5*10^{-8}$	0.9	28	$6*10^{-3}$	$1*10^{-6}$	0.9
	random	5	$8*10^{-3}$	1.0	132	$4*10^{-3}$	$3*10^{-7}$	0.8
100 pM	aptamer	58	$1*10^{-7}$	0.8	33	$6*10^{-3}$	$1*10^{-6}$	0.9
	random	6	$2*10^{-2}$	0.8	132	$4*10^{-3}$	$3*10^{-7}$	0.8
1 nM	aptamer	56	$3*10^{-7}$	0.7	39	$6*10^{-3}$	$4*10^{-6}$	0.8
	random	4	$1*10^{-2}$	0.9	135	$4*10^{-3}$	$3*10^{-7}$	0.8
10 nM	aptamer	53	$4*10^{-7}$	0.7	45	$6*10^{-3}$	$5*10^{-6}$	0.7
	random	5	$1*10^{-2}$	0.9	135	$4*10^{-3}$	$4*10^{-7}$	0.8
100 nM	aptamer	44	$5*10^{-6}$	0.7	52	$6*10^{-3}$	$9*10^{-8}$	0.8
	random	6	$1*10^{-2}$	0.9	136	$4*10^{-3}$	$4*10^{-7}$	0.8
1 μM	aptamer	37	$9*10^{-6}$	0.7	62	$6*10^{-3}$	$4*10^{-7}$	0.7
	random	5	$1*10^{-2}$	0.9	136	$4*10^{-3}$	$4*10^{-7}$	0.8
10 μM	aptamer	30	$1*10^{-5}$	0.7	70	$6*10^{-3}$	$6*10^{-7}$	0.7
	random	6	$1*10^{-2}$	0.9	137	$4*10^{-3}$	$7*10^{-7}$	0.7
100 μM	aptamer	52	$2*10^{-5}$	0.7	72	$6*10^{-3}$	$7*10^{-7}$	0.7
	random	38	$1*10^{-7}$	1.0	109	$4*10^{-3}$	$6*10^{-7}$	0.7

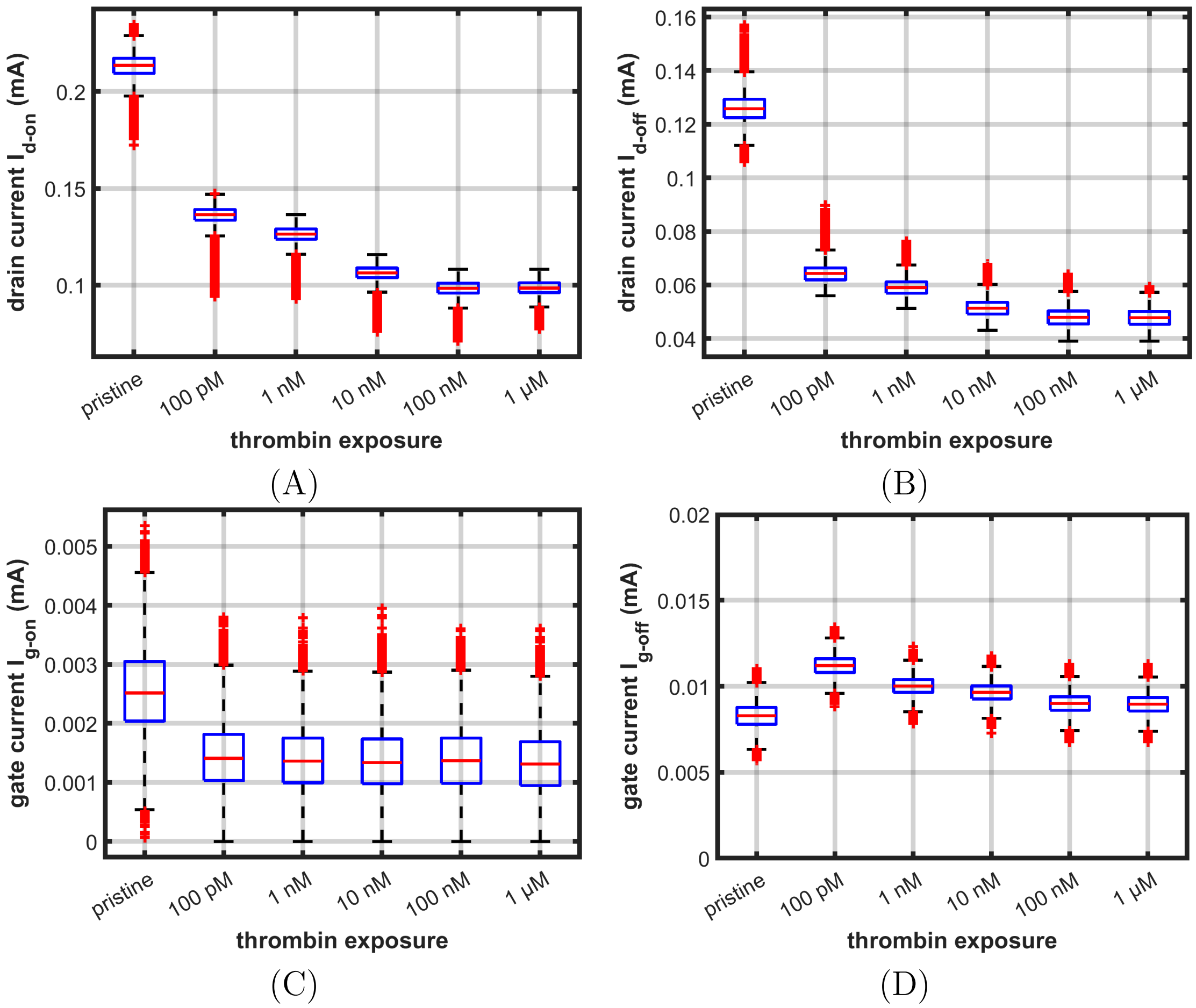

Figure S15: **Box-whisker plot of LOECT-based aptasensor transient characteristic at different concentration of thrombin exposure**. The measured data from Figure 4**A** for drain current I_{d} and Figure S14 for gate current I_{g} are shown in (**A** and **B**) for I_{d} and (**C** and **D**) for I_{g} for the LOECT-based aptasensor with the gate to channel area ratio 1:1 at both (**A** and **C**) on- and (**B** and **D**) off-state. The red horizontal lines within the boxes represent the medians of the distributions; the boxes denote interquartile ranges (IQRs); the error bars correspond to the values furthest from the boxes, up to a distance of 1.5 times the IQR; the points lying beyond these intervals are considered as outliers.

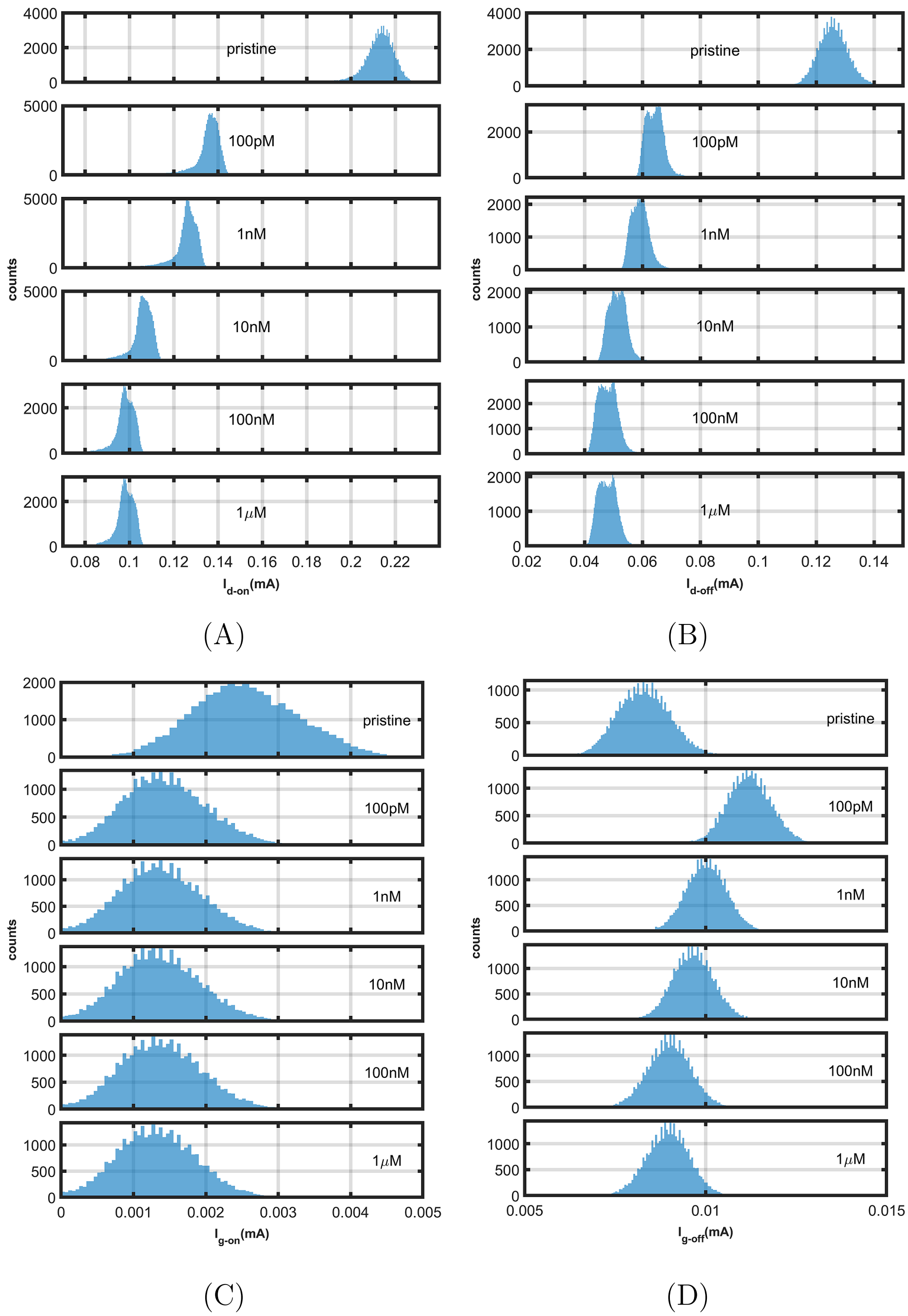

Figure S16: **Histograms of LOECT-based aptasensor transient characteristic at different concentration of thrombin exposure** The measured data from Figure 4**A** for I_{d} and Figure S14 for I_{g} are shown in (**A** and **B**) for I_{d} and (**C** and **D**) for I_{g} for the LOECT-based aptasensor with the gate to channel area ratio is 1:1 at both (**A** and **C**) on- and (**B** and **D**) off-state. A systematic progression of the $I_{\text{d-on}}$, $I_{\text{d-off}}$, and $I_{\text{g-off}}$ with increasing concentration is found, including the pristine sample for the $I_{\text{d-on}}$ and $I_{\text{d-off}}$, but excluding it for the $I_{\text{g-off}}$.

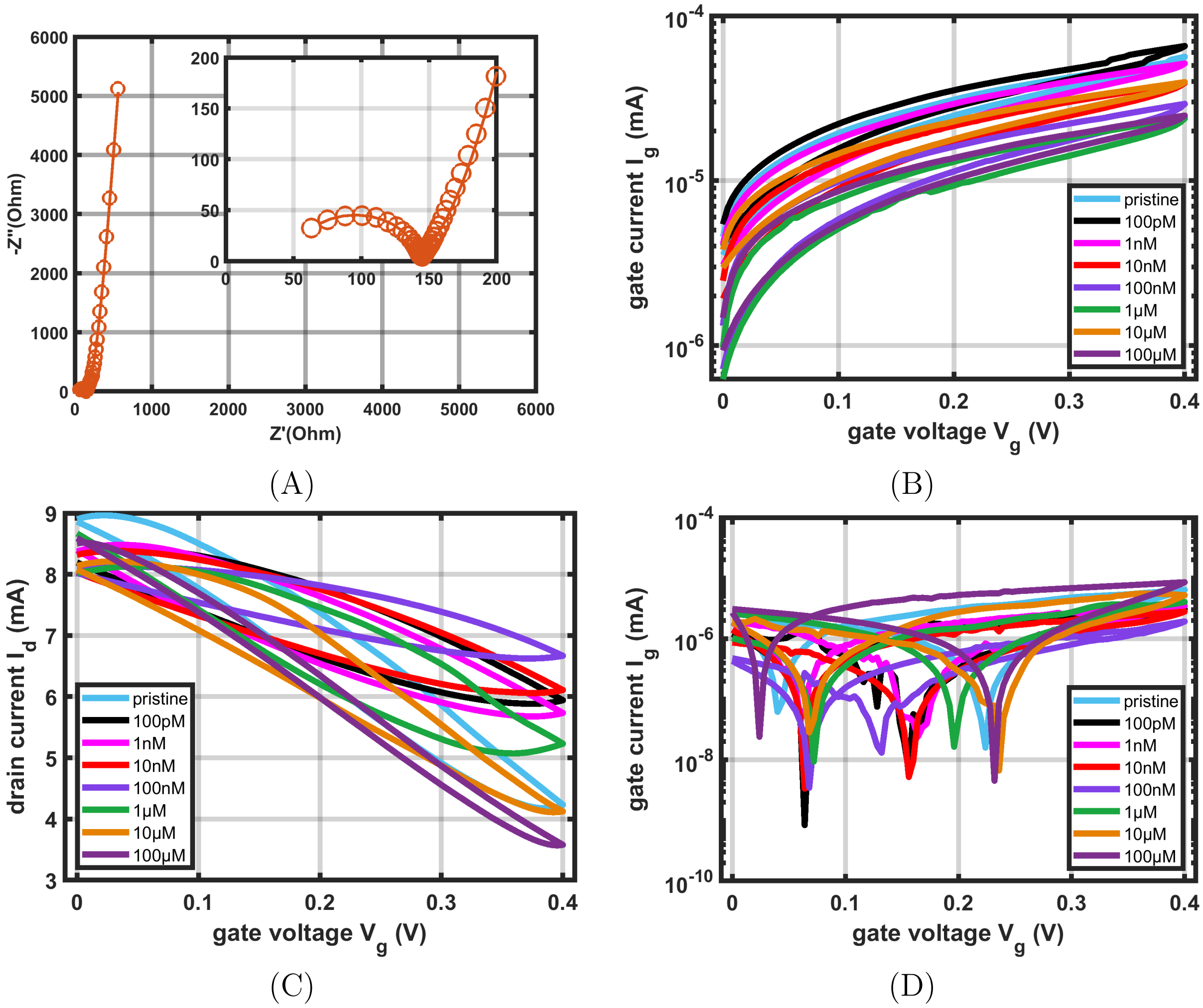

Figure S17: **EIS of electropolymerized PEDOT:PSS channel and interrogation of VOECT-based aptasensor**. (**A**) EIS of electropolymerized PEDOT:PSS film with volume $2*10^{-3}$ mm^3, showing a semicircle at high frequency (cf. inset), suggesting a charge transport, and Warburg process at low frequency. The spectrum is fitted with an equivalent circuit in Figure S7**A** and presented in table S3. Transfer characteristic of VOECT with adsorbed (**B**) TBA-29 and (**C** and **D**) random 29-mer oligo on the gold gate electrode. The gate electrode area, PEDOT:PSS channel width, and length, are 0.15 mm^2, 315 μm, and 300 nm, respectively. The relation between (**C**) I_d vs V_g and (**B** and **D**) I_g vs V_g are shown at different concentration of thrombin exposure in electrolyte. The drain voltage V_d is kept constant at -0.2 V

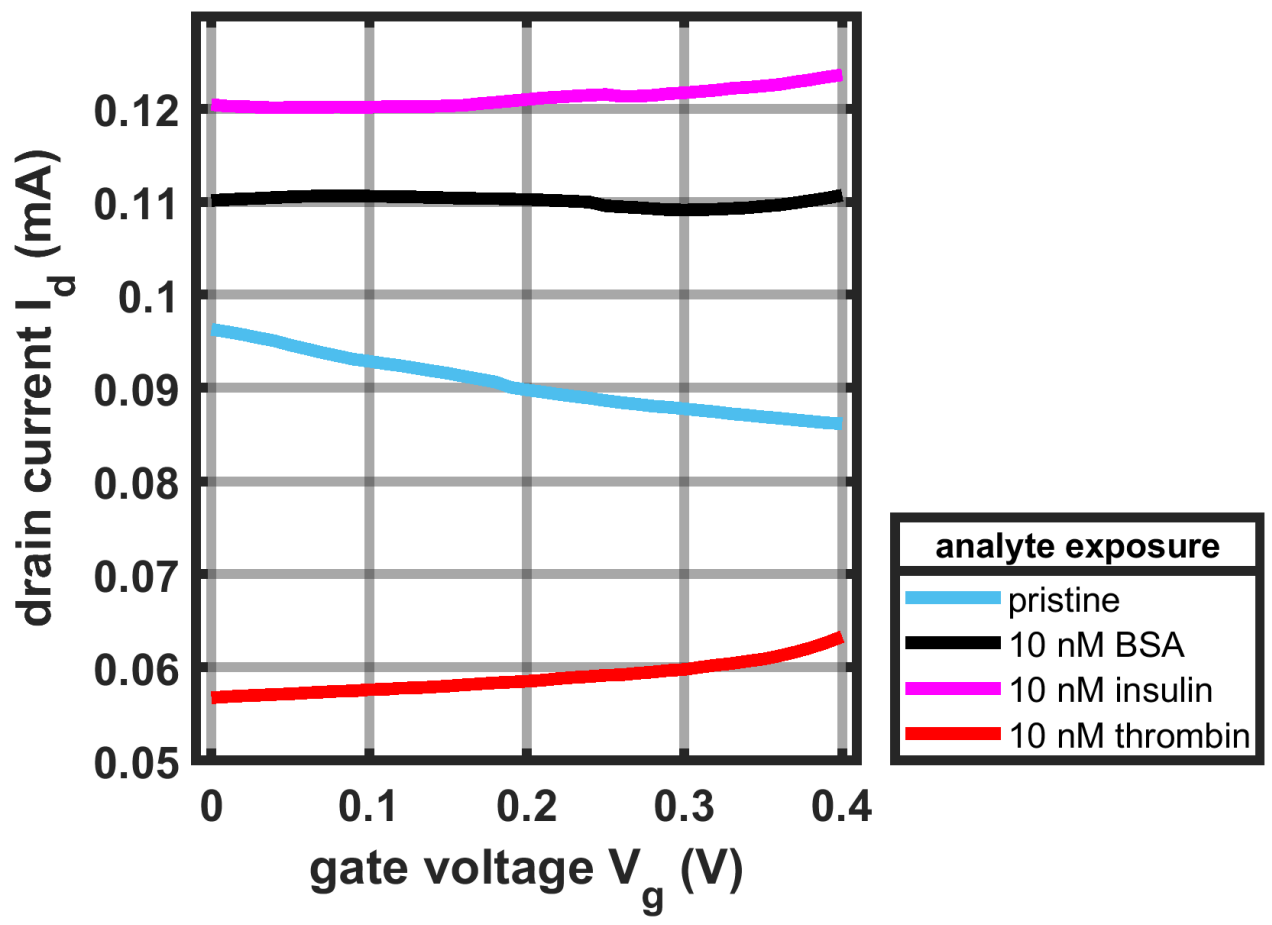

(A)

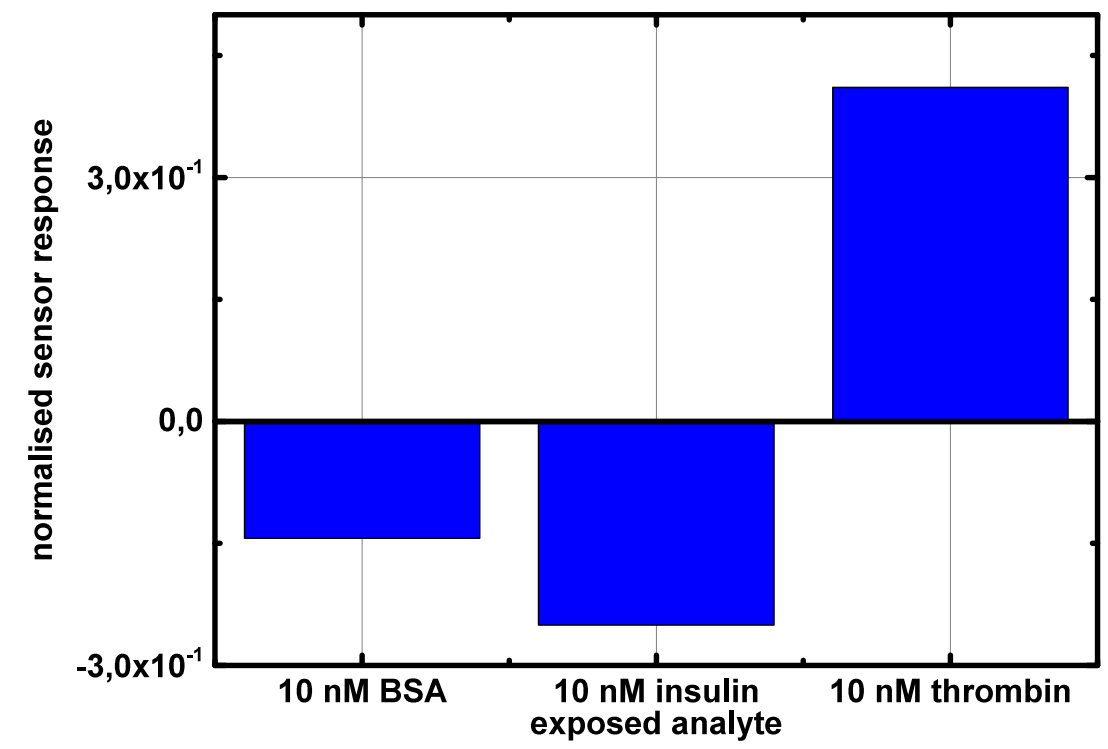

(B)

Figure S18: **Selectivity test of an LOECT-based aptasensor for thrombin** (**A**) An LOECT-based aptasensor for thrombin with gate to channel area ratio 1:1 is exposed and tested consecutively against bovine serum albumin (BSA), insulin, and thrombin, each with incubation period 30 minutes and a washing step in between. The aptasensor exhibits a clear selectivity, since the OECT drain current I_d only drops after the sensor is exposed to thrombin. (**B**) The normalized response of the LOECT-based aptasensor in Figure S18**A** is calculated according to Equation 3 and presented as a bar plot.

Table S2: **Comparison of different electrochemical methods for thrombin detection in literature**.

aptamer	method	linear range	LOD	references
TBA-15 and TBA-29	DPV	1 fM-6 pM	142.9 aM	[1]
TBA-15 and TBA-29	DPV	1 pM-1 nM	560 fM	[2]
TBA-15	DPV	100 pM-25 nM	20 pM	[3]
TBA-15	DPV	50 fM-60 nM	15 fM	[4]
TBA-15	DPV	2 pM-10 nM	670 fM	[5]
TBA-15	DPV	50 pM-50 nM	16.67 pM	[5]
TBA-15	EIS	100 fM-20 nM	38 nM	[6]
TBA-15	CV	1 pM-30 nM	560 fM	[7]
TBA-15	DPV	1 pM-30 nM	320 fM	[8]
TBA-15	Chronoamperometry	100 fM-20 nM	23 fM	[9]
TBA-15	DPV	100 pM-10 nM	70 pM	[10]
TBA-15	DPV	200 fM-20 nM	20 fM	[11]
TBA-15	DPV	7.3 pM-7.3 nM	4.6 pM	[12]
TBA-29	DPV	1 pM-10 μM	143 fM	[13]
TBA-15	OECT		5 pM	[14]

Table S3: **Electrochemical circuit analysis for PEDOT:PSS channel**. Parameter of equivalent electrochemical circuit in Figure S7**A** to model the EIS in Figure S6**D** and S17**A**, for the spin-coated and electropolymerized PEDOT:PSS film, respectively. C2 is used to calculate the volumetric capacitance of spin-coated (50 F/cm^3) and electropolymerized (150 F/cm^3) PEDOT:PSS film.

PEDOT:PSS channel	channel volume (mm^3)	Rs (Ω)	R1 (Ω)	C1 (F)	R2 (Ω)	W1 $\left(\frac{\sqrt{s}}{\Omega}\right)$	C2 (F)
spincoated	0.002	40	60	$8*10^{-8}$	$3*10^{5}$	0.007	$1*10^{-4}$
electropolymerized	0.002	50	90	$4*10^{-8}$	$5*10^{5}$	0.002	$3*10^{-4}$

Table S4: **Overview of statistical analysis of OECT-based aptasensor drain current I_d with pulsed gate voltage V_g**. The drain current at both (a) on- ($I_{d\text{-}on}$) and (b) off state ($I_{d\text{-}off}$) at varying thrombin concentration from 0 (pristine) until 1 μM are taken from Figures 4**A**, S15**A-B**, and S16**A-B**.

	$I_{d\text{-}on}$ (mA)			
**thrombin exposure**	**mean**	**standard deviation**	**median**	**median absolute deviation**
**pristine**	0.213	0.007	0.213	0.005
**100 pM**	0.135	0.007	0.136	0.007
**1 nM**	0.125	0.006	0.126	0.006
**10 nM**	0.106	0.005	0.106	0.005
**100 nM**	0.098	0.005	0.098	0.005
**1 μM**	0.098	0.004	0.098	0.005
	**$I_{d\text{-}off}$ (mA)**			
**thrombin exposure**	**mean**	**standard deviation**	**median**	**median absolute deviation**
**pristine**	0.126	0.005	0.126	0.006
**100 pM**	0.064	0.003	0.064	0.004
**1 nM**	0.059	0.003	0.059	0.004
**10 nM**	0.051	0.003	0.051	0.004
**100 nM**	0.048	0.003	0.048	0.004
**1 μM**	0.048	0.003	0.048	0.004

Table S5: **Overview of statistical analysis of OECT-based aptasensor gate current I_g with pulsed gate voltage V_g.** The gate current at both (a) on- ($I_{g\text{-on}}$) and off state ($I_{g\text{-off}}$) at varying thrombin concentration from 0 (pristine) until 1 μM from Figures S14, S15 **C-D**, and S16 **C-D**.

	$I_{g\text{-on}}$ (mA)			
**thrombin exposure**	**mean**	**standard deviation**	**median**	**median absolute deviation**
**pristine**	0.0026	0.00073	0.0025	0.0006
**100 pM**	0.0014	0.00057	0.0014	0.0007
**1 nM**	0.0014	0.00055	0.0014	0.0007
**10 nM**	0.0014	0.00056	0.0013	0.0007
**100 nM**	0.0014	0.00055	0.0014	0.0007
**1 μM**	0.0013	0.00054	0.0013	0.0006
	$I_{g\text{-off}}$ (mA)			
**thrombin exposure**	**mean**	**standard deviation**	**median**	**median absolute deviation**
**pristine**	0.0083	0.0007	0.0083	0.0008
**100 pM**	0.0112	0.00059	0.0112	0.0007
**1 nM**	0.01	0.00055	0.01	0.0007
**10 nM**	0.0096	0.00056	0.0096	0.0007
**100 nM**	0.009	0.00058	0.009	0.0007
**1 μM**	0.009	0.0058	0.009	0.0007

References

(1) Chen, Y.; Xiang, J.; Liu, B.; Chen, Z.; Zuo, X. Gold nanoparticle-engineered electrochemical aptamer biosensor for ultrasensitive detection of thrombin. *Analytical Methods* **2020**, *12*, 3729–3733.

(2) Li, W.; Zhao, D.; Tian, D.; Zhai, M.; Xu, H.; Zheng, L.; Li, S.; Sang, Y. Electrochemical aptasensor based on proximity binding-induced DNA networked for enzyme-free and ultrasensitive detection of thrombin. *Journal of Electroanalytical Chemistry* **2021**, *895*, 115447.

(3) Zheng, Y.; Yuan, Y.; Chai, Y.; Yuan, R. A label-free electrochemical aptasensor based on the catalysis of manganese porphyrins for detection of thrombin. *Biosensors and Bioelectronics* **2015**, *66*, 585–589.

(4) Wu, Y.; Zou, L.; Lei, S.; Yu, Q.; Ye, B. Highly sensitive electrochemical thrombin aptasensor based on peptide-enhanced electrocatalysis of hemin/G-quadruplex and nanocomposite as nanocarrier. *Biosensors and Bioelectronics* **2017**, *97*, 317–324.

(5) Yang, P.; Guo, X.; Zhang, J.; Chen, C.; Gan, Y.; Xie, W.; Du, Y.; Wu, Z. Picomolar thrombin detection by orchestration of triple signal amplification strategy with hierarchically porous Ti3C2Tx MXene electrode material-catalytic hairpin assembly reaction-metallic nanoprobes. *Biosensors and Bioelectronics* **2022**, *208*, 114228.

(6) Xu, H.; Zhang, T.; Gu, Y.; Yan, X.; Lu, N.; Liu, H.; Xu, Z.; Xing, Y.; Song, Y.; Zhang, Z.; Yang, M. An electrochemical thrombin aptasensor based on the use of graphite-like C3N4 modified with silver nanoparticles. *Microchimica Acta* **2020**, *187*, 163.

(7) Bai, L.; Yan, B.; Chai, Y.; Yuan, R.; Yuan, Y.; Xie, S.; Jiang, L.; He, Y. An electrochemical aptasensor for thrombin detection based on direct electrochemistry of glucose

oxidase using a functionalized graphene hybrid for amplification. *Analyst* **2013**, *138*, 6595–6599.

(8) Yang, X.; Lv, J.; Yang, Z.; Yuan, R.; Chai, Y. A Sensitive Electrochemical Aptasensor for Thrombin Detection Based on Electroactive Co-Based Metal–Organic Frameworks with Target-Triggering NESA Strategy. *Analytical Chemistry* **2017**, *89*, 11636–11640.

(9) Chen, S.; Liu, P.; Su, K.; Li, X.; Qin, Z.; Xu, W.; Chen, J.; Li, C.; Qiu, J. Electrochemical aptasensor for thrombin using co-catalysis of hemin/G-quadruplex DNAzyme and octahedral $Cu_2$2O-Au nanocomposites for signal amplification. *Biosensors and Bioelectronics* **2018**, *99*, 338–345.

(10) Ahour, F.; Ahsani, M. An electrochemical label-free and sensitive thrombin aptasensor based on graphene oxide modified pencil graphite electrode. *Biosensors and Bioelectronics* **2016**, *86*, 764–769.

(11) Tian, R.; Chen, X.; Li, Q.; Yao, C. An electrochemical aptasensor electrocatalyst for detection of thrombin. *Analytical Biochemistry* **2016**, *500*, 73–79.

(12) Fan, H.; Li, H.; Wang, Q.; He, P.; Fang, Y. A host–guest-recognition-based electrochemical aptasensor for thrombin detection. *Biosensors and Bioelectronics* **2012**, *35*, 33–36.

(13) Bai, H.-Y.; Campo, F. J. D.; Tsai, Y.-C. Sensitive electrochemical thrombin aptasensor based on gold disk microelectrode arrays. *Biosensors and Bioelectronics* **2013**, *42*, 17–22.

(14) Peruzzi, C.; Battistoni, S.; Montesarchio, D.; Cocuzza, M.; Marasso, S. L.; Verna, A.; Pasquardini, L.; Verucchi, R.; Aversa, L.; Erokhin, V.; D'Angelo, P.; Iannotta, S. Interfacing aptamers, nanoparticles and graphene in a hierarchical structure for highly selective detection of biomolecules in OECT devices. *Scientific Reports* **2021**, *11*, 9380.
